# The low overpotential regime of acidic water oxidation part I: the importance of O_2_ detection†

**DOI:** 10.1039/d1ee03914h

**Published:** 2022-03-19

**Authors:** Soren B. Scott, Reshma R. Rao, Choongman Moon, Jakob E. Sørensen, Jakob Kibsgaard, Yang Shao-Horn, Ib Chorkendorff

**Affiliations:** SurfCat Section for Surface Physics and Catalysis, Department of Physics, Technical University of Denmark Kgs. Lyngby Denmark ibchork@fysik.dtu.dk; Department of Mechanical Engineering, Massachusetts Institute of Technology Cambridge Massachusetts USA reshma.rao@imperial.ac.uk

## Abstract

The high overpotential required for the oxygen evolution reaction (OER) represents a significant barrier for the production of closed-cycle renewable fuels and chemicals. Ruthenium dioxide is among the most active catalysts for OER in acid, but the activity at low overpotentials can be difficult to measure due to high capacitance. In this work, we use electrochemistry – mass spectrometry to obtain accurate OER activity measurements spanning six orders of magnitude on a model series of ruthenium-based catalysts in acidic electrolyte, quantifying electrocatalytic O_2_ production at potential as low as 1.30 V_RHE_. We show that the potential-dependent O_2_ production rate, *i.e.*, the Tafel slope, exhibits three regimes, revealing a previously unobserved Tafel slope of 25 mV decade^−1^ below 1.4 V_RHE_. We fit the expanded activity data to a microkinetic model based on potential-dependent coverage of the surface intermediates from which the rate-determining step takes place. Our results demonstrate how the familiar quantities “onset potential” and “exchange current density” are influenced by the sensitivity of the detection method.

## Abstract 2

By measuring O_2_ with high sensitivity, we open a mechanistic window into water oxidation on RuO_*x*_ in acid.

## Graphical abstract

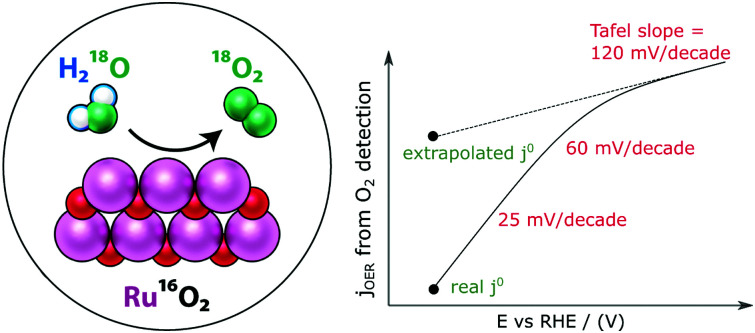

**Broader context**

Halving net CO_2_ emissions by 2030 and eliminating them by 2050, as is necessary to limit global warming to 1.5 °C, will require hundreds of gigawatts capacity of renewable energy storage and conversion of electrical to chemical energy, most of which is expected to rely on water electrolysis. The slow kinetics of the oxygen evolution reaction (OER) is a main source of energy loss in polymer electrolyte membrane (PEM) and alkaline water electrolysis as well as emerging electrochemical technologies such as CO_2_ reduction and metal–air batteries, and thus a hindrance in their uptake. Catalyzing OER is a particular challenge in the acidic electrolyte of the otherwise most advantageous PEM electrolyzers, where only oxides of scarce elements iridium and ruthenium are sufficiently stable and active as OER catalysts. Attempts to design more active OER catalysts rely on understanding the electrocatalytic mechanism to enhance the kinetics. However, uncertainties remain about the electrocatalytic mechanism, particularly the activity at low overpotential, since quantitatively probing sub monolayers of evolved oxygen is a challenge. In this work, we use ultrasensitive O_2_ detection to accurately measure OER activity of ruthenium oxides at overpotentials down to 70 mV and thus provide new insights into the OER mechanism.

## Introduction

Electrochemical water oxidation is a key enabler to the renewable production of energy carriers such as hydrogen^1–3^ and chemicals such as ethanol and ethylene from carbon dioxide reduction^4,5^ and ammonia from nitrogen reduction.^6–8^ However, the slow kinetics of water oxidation is limiting the large-scale uptake of electrosynthesis of fuels and chemicals.^9–11^ Particularly, in proton exchange membrane electrolyzers that operate at low pH, only oxides of the rare noble metals iridium and ruthenium exhibit reasonable activity^3,12,13^ and stability.^14–16^ Design of oxygen evolution catalysts with enhanced stability and specific activity is needed,^10,17,18^ which would lower the noble metal loading required for water oxidation and reduce the capital cost of electrolyzers. This need in turn requires more knowledge of the mechanisms of water oxidation electrocatalysis. In this work, we focus on ruthenium oxide electrocatalysts because they are the most active in acidic electrolyte^19,20^ and because RuO_2_ has been extensively studied as a model electrocatalyst.^21–23^

Computational methods have been used extensively to predict trends in the electrocatalytic activity by calculating the energies of electrocatalytic intermediates.^24–26^ In a highly influential work,^27^ Rossmeisl and coworkers assumed a metal peroxide pathway involving adsorbate oxidation *via* the electrocatalytic intermediates *, *OH, *O, and *OOH (where * represents a metal atom at the surface of the metal oxide). In this model, the correlation (scaling) of the adsorption energies of *OH and *OOH is used to explain why an overpotential of ∼200–300 mV relative to the nominal equilibrium potential of 1.23 V_RHE_ (RHE as reversible hydrogen electrode) is required to drive the OER on the theoretically predicted optimal catalyst.^27^ The lowest theoretical overpotential at which no steps are uphill in free energy is called the limiting overpotential.^27^ Specifically for RuO_2_ surfaces, the limiting overpotential ranges from 380 mV for the (110) facet to 340 mV for the (100) facet.^22^

Unfortunately, the direct correspondence of the limiting potential to experimental observations has been elusive^28–30^ because of several factors. On the theoretical side, translating the limiting potential to an actual OER rate requires the development of kinetic models of the OER, which rely on identifying and accurately computing reaction barriers in the complex multi-step reaction, a computationally challenging task.^31–33^ On the experimental side, determining the physical origin of anodic current at low oxygen-evolution overpotentials (<1.45 V_RHE_), and thus the true oxygen evolution activity, is challenging, not least on ruthenium oxides, for three reasons: First, RuO_*x*_-based materials have large capacitance, which contributes significantly to the oxidation current.^34^ The capacitance is further amplified for amorphous RuO_*x*_ and hydrous RuO_*x*_,^35,36^ which can show larger gravimetric oxygen evolution activity compared to crystalline oxides.^37^ Secondly, recent studies by Cherevko *et al.*^38^ and Hodnik *et al.*^39^ have alluded to the possibility of electrochemical dissolution of Ru ions in solution preceding the onset of OER, further raising questions about the onset of oxygen evolution. These observations highlight that the concept of “onset potential”, measured experimentally, is ambiguous because it depends on the ability to reliably measure OER activity among other possible sources of anodic current. Thirdly, detection of O_2_ has not been sensitive or quantitative enough to provide an accurate independent measurement of the OER rate at low overpotentials. On single crystal RuO_2_(110) in acid at potentials lower than 1.5 V_RHE_, OER current densities of ∼5 μA cm^−2^_oxide_ and lower have been observed, which corresponds to an oxygen flux of ∼10^−10^ mol s^−1^ cm^−2^_oxide_ and lower, which is below the detection limit of conventional electrocatalytic O_2_ detection by differential electrochemistry mass spectrometry (DEMS).^40^ An emerging, fundamental question is whether OER can be observed at overpotentials less than 1.5 V_RHE_.

The dependence of OER activity on electrochemical potential is often evaluated as the Tafel slope, defined as the change in overpotential required to produce an order of magnitude increase in water oxidation current density. Microkinetic models predict potential-dependent Tafel slopes for metal oxides due to an increase in the coverage of species participating in the rate-determining step with increasing potential.^31,41–43^ A recent theoretical study on RuO_2_(110) has suggested that the rate-determining step is the O–O bond formation, *O + H_2_O → *OOH + H^+^ + e^−^, from which a Tafel slope of ∼39 mV decade^−1^ can be obtained at potentials lower than ∼1.5 V_RHE_, where the active coordinatively unsaturated Ru sites (*) is filled with *OH.^31^ The Tafel slope was proposed to transition to 120 mV decade^−1^ at potentials higher than ∼1.5 V_RHE_ where the *O covered surface was thermodynamically stable.^31^ Experimentally, at potentials greater than ∼1.55 V_RHE_, a Tafel slope of ∼120 mV decade^−1^ has been noted on RuO_2_ nanoparticles^19^ and oriented thin films, and from ∼1.45 to ∼1.55 V_RHE_,^44,45^ a Tafel slope of ∼60 mV decade^−1^ has been observed. Therefore, corroborating theoretical predictions^31^ of possible Tafel slopes lower than 60 mV decade^−1^ at potentials lower than ∼1.5 V_RHE_ is challenging. Accurate measurement of OER activity at low overpotential could be extremely valuable in informing the computational results, considering that this potential dependence of the activity is dependent on the computed energetics of the reaction intermediates.^22,41,42,46^

Recent developments in microchip-based electrochemistry-mass spectrometry (chip EC-MS) technology^40^ has for the first time enabled real time, quantitative detection of gases in amounts as low as ∼10^−12^ mol s^−1^. This enables the ultra-sensitive detection of electrochemically produced O_2_ and thus accurate measurement of OER activity in spite of the challenges highlighted above. This EC-MS setup allows for 100% collection efficiency and well-characterized reproducible mass transport, allowing quantitative real-time detection of gaseous electrochemical desorption products at sub-picomol per second sensitivity,^47,48^ contributing to the unravelling of the mechanisms of electrocatalytic hydrogen evolution,^48^ CO reduction,^49^ propene oxidation,^50,51^ and CO oxidation.^47,52,53^ It has been used previously for isotope-labeling experiments in the OER,^53,54^ but its sensitivity has not previously been utilized to push the limits of accurate activity measurements. In this work, we use chip EC-MS to study the kinetics of water oxidation on RuO_2_ surfaces at potentials <1.50 V_RHE_. The lowest overpotential at which we could consistently detect sustained O_2_ production (at about 0.25 pmol s^−1^) was 1.30 V_RHE_, only 70 mV above the standard thermodynamic potential for water oxidation. RuO_2_ films sputter deposited at temperatures ranging from room temperature to 400 °C were found to have a Tafel slope of ∼25 mV decade^−1^ below 1.45 V_RHE_, which increased to 60 mV decade^−1^ up to 1.55 V_RHE_ and transitioned to 120 mV decade^−1^ at higher potentials. We demonstrate that improving sensitivity towards oxygen generation can bypass the so-called “onset potential” observed in less sensitive methods, enabling accurate study of the mechanistically interesting low-overpotential regime by actually measuring the O_2_ evolution. This article is the first in a two-part series, where the second addresses electrocatalyst stability and employs isotope-labeling to address remaining mechanistic questions.

## Results and discussion

### Detecting oxygen evolution at low overpotentials

**Fig. 1:**
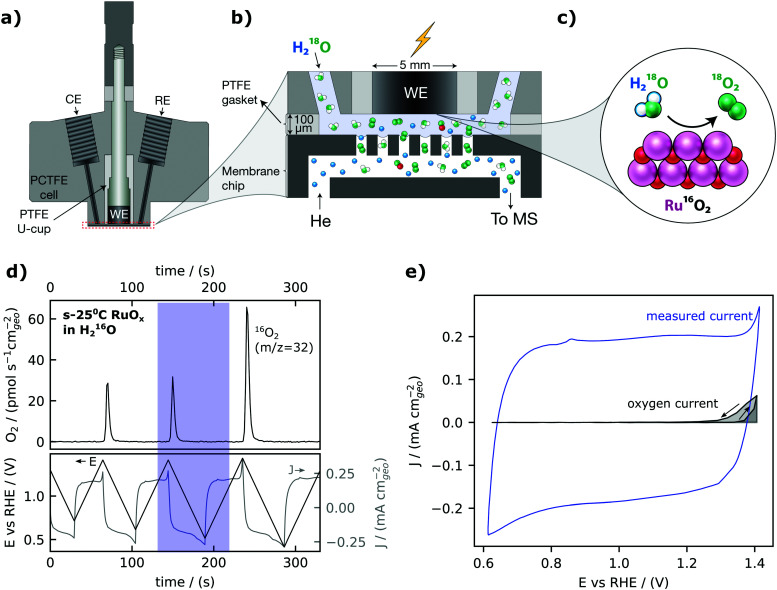
Electrochemistry-Mass spectrometry. (a) Schematic of the EC-MS cell. (b). Zoom-in on the working volume of the EC-MS setup. (c) Schematic diagram of oxygen evolution (here with isotopically labelled electrolyte). (d) EC-MS plot of cyclic voltammetry of an amorphous RuO_*x*_ film sputter-deposited at 25 °C (s-25 °C RuO_*x*_) in He-saturated 0.1 M HClO_4_ at a scan rate of 20 mV s^−1^. The data from the blue highlight, a single cycle between 0.62 V_RHE_ and 1.40 V_RHE_ is used for (e), the cyclic voltammogram with a highlight indicating the portion of the charge associated with oxygen evolution, determined by integration of the calibrated MS signal and application of Faraday's law of electrolysis. We note that the diffusion time for oxygen (3 seconds or 60 mV at a scan rate of 20 mV s^−1^) was considered while determining the oxygen current. Charging current is clearly much higher than OER current at low OER overpotentials.

Our electrochemistry – mass spectrometry (EC-MS) setup for detection of electrochemically generated O_2_ is sketched in Fig. 1a–c and described in detail in reference.^40^ We used a cell made from a resistive polymer, PTCFE, to hold a working electrode disk, reference electrode (RE) and counter electrode (CE) in place. The interface to the mass spectrometer, which is at ∼10^−6^ mbar high vacuum, is formed in a silicon microchip: a perforated membrane allows the external electrochemical environment to equilibrate with an internal volume etched into the chip which functions as a nanoscopic head-space, saturating the electrolyte with inert gas (helium, He) and carrying gaseous electrochemical products through a capillary fabricated into the chip to the vacuum chamber. A gasket of Teflon is used together with the cell to define a 100 μm thick layer of electrolyte between the electrode and the chip. Sputter-deposited RuO_*x*_ films, as the working electrode, were prepared by reactive magnetron sputtering from a Ru target under a 3 mTorr pressure (80% argon and 20% oxygen) on a glassy carbon electrode at room temperature, 200 °C, 300 °C and 400 °C– hereafter also referred to as s-25 °C RuO_*x*_, s-200 °C RuO_*x*_, s-300 °C RuO_*x*_ and s-400 °C RuO_*x*_ respectively. The crystallinity increases with increasing deposition temperature, with the room temperature films being XRD-amorphous and the 400 °C films showing the rutile structure (Fig. S1, ESI†). The surface roughness is comparable across all films from Table S1 (ESI†).

The calibrated mass spectrometer signal for O_2_ (*m*/*z* = 32) and corresponding current and voltage collected from cyclic voltammetry of a room-temperature sputter-deposited RuO_*x*_ film are shown in Fig. 1d. At potentials less than 1.40 V_RHE_, the current expected from the rate of OER measured from mass spectrometry (in grey, Fig. 1e) is much smaller than the measured current from cyclic voltammetry (in blue, Fig. 1e). The large difference can be attributed to faradaic pseudocapacitive currents, RuO_*x*_(OH)_*y*_ + *δ*H^+^ + *δ*e^−^ ↔ RuO_*x*−*δ*_(OH)_*y*+*δ*_,^55,56^ which highlights the challenges of detecting OER at low overpontentials using electrochemical techniques alone. Therefore, the small magnitude of the water oxidation currents relative to the capacitive currents in the cyclic voltammogram illustrate that oxygen detection is essential when measuring OER at low overpotential on high-capacitive materials such as RuO_2_.

**Fig. 2:**
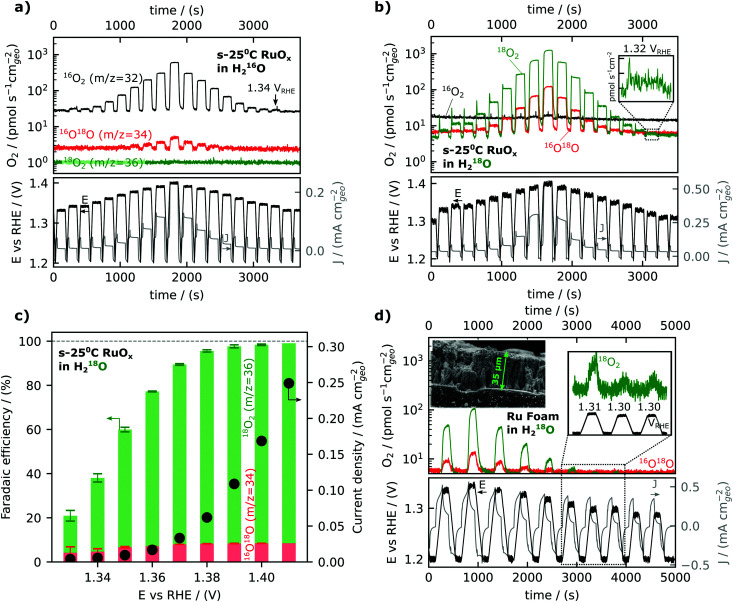
Activity as measured by EC-MS. (a) calibrated signals from OER on amorphous RuO_2_ for the three O_2_ isotopes in un-labelled electrolyte. The small amount of *m*/*z* = 34 signal is due to the natural abundance of ^18^O. (b) The same experiment in ^18^O labelled electrolyte, showing a lower baseline and thus more sensitivity. (c) Faradaic efficiencies (bars) and total measured current densities (dots, right *y*-axis) for the experiment in (b). Faradaic efficiencies were calculated from the measured average current density and O_2_ signals in the last 30 seconds of a two-minute potential hold. Error bars represent the standard deviation of apparent instantaneous faradaic efficiency during the same 30 seconds. (d) Calibrated signals from OER on Ru foam for the three O_2_ isotopes in an ^18^O labelled electrolyte. The small amount of *m*/*z* = 34 signal is consistent with the purity of labelled electrolyte (0.1 M HClO_4_ in 97% H_2_^18^O labelled water). Ru foam samples were synthesized by ruthenium cathodic electrodeposition at −6 V_RHE_ from a solution of 10 mM RuCl_3_ under vigorous hydrogen evolution conditions.

We will now turn to steady-state oxygen generation measured by mass signal at *m*/*z* = 32 detected in He-saturated 0.1 M HClO_4_ electrolyte as low as 1.34 V_RHE_, as shown in Fig. 2a. The potential was scanned at 5 mV s^−1^ in 0.1 M HClO_4_ from open circuit (∼0.8–0.9 V_RHE_) to a resting potential of 1.2 V_RHE_. The potential was then ramped to the working potential at which potentiostatic measurements were conducted for 2 minutes. Following the 2 minutes at the working potential, the potential was ramped backed down to the resting potential of 1.2 V_RHE_ The mass signal observed for *m*/*z* = 32 and *m*/*z* = 34 (due to the natural abundance of ^18^O) on a representative film sputter deposited at room temperature is shown to track the potential step change in Fig. 2a, demonstrating the detection of oxygen generation as a function of voltage. The reproducibility of data and analogous data for films prepared at different temperatures can be found in the ESI† and accompanying database. Oxygen was detected at each working potential, down to 1.34 V_RHE_, and the oxygen signal became masked by the background of the ^16^O_2_ (*m*/*z* = 32) at potentials lower than 1.34 V_RHE_.

An equivalent experiment in an isotopically labelled electrolyte (He-saturated 0.1 M HClO_4_ in 97% H_2_^18^O labelled water) is shown in Fig. 2b, showing a clear oxygen signal at 1.32 V_RHE_ or a nominal overpotential of 90 mV. Isotopically labeling the water in the electrolyte with oxygen-18 can achieve higher sensitivity and allow the detection of oxygen evolution at lower overpotentials, due to the lower background of *m*/*z* = 36 (^18^O_2_) compared to *m*/*z* = 32 (^16^O_2_). The ^16^O^18^O/^18^O_2_ ratio is not significantly different than the expected value of ∼6% due to the ^16^O impurity in the labelled electrolyte at any potential, *i.e.*, no significant lattice oxygen evolution, involving ^16^O of the oxides, was observed in this experiment. Small lattice oxygen evolution signals (<0.1% of the total O_2_ evolved) were observed in more sensitive experiments using labelled Ru^18^O_*x*_ films, which is the subject of the second article of this series (DOI: 10.1039/D1EE03915F).

The faradaic efficiency for oxygen evolution was found to be ∼100% at potentials greater than 1.40 V_RHE_ but dropped at lower overpotential, reaching ∼ 20% at 1.33 V_RHE_, as shown in Fig. 2c. The faradaic efficiency is here defined as the ratio of the OER-related current density obtained by converting the oxygen flux detected by EC-MS to the total current density measured. The oxygen flux is averaged over the final 30 seconds of the 2-minute potentiostatic measurements at the working potential. The exponential decrease in the current density of oxygen evolution (black dots in Fig. 2c) from ∼1.42 V_RHE_ to ∼1.32 V_RHE_ was accompanied with significant reduction in the faradaic efficiency for OER from ∼1.42 V_RHE_ to ∼1.32 V_RHE_, during which the residual capacitance current became dominant. Notably, at potentials greater than 1.40 V_RHE_, the oxidation current comes from OER (faradaic efficiency ∼100%), and thus we can use current alone to compare OER activity.

While the activity measurements exemplified in Fig. 2a and b were conducted in electrolyte saturated with He, and thus differed from the standard equilibrium condition of 1 bar O_2_, separate experiments showed that this did not affect the measured activity. In chip EC-MS, the electrolyte is saturated through the chip with a carrier gas, here He, while product gases such as O_2_ diffuse through the working volume of the electrolyte and escape through the chip. The concentration profile of a light gas such as O_2_ in the electrolyte can be estimated by Fick's first law of diffusion, and so depends on the production rate.^47^ At our detection limit of about 1 pmol s^−1^ cm^−2^, the concentration at the electrode surface is about 50 pM, or 4 × 10^−8^ of the saturation concentration, resulting in a Nernst equilibrium potential of 1.12 V_RHE_ (calculation details in Section 2 of the ESI†). Thus, the actual thermodynamic overpotential for OER is larger than the nominal overpotential based on the standard equilibrium potential of 1.23 V_RHE_. However, using isotopically labeled electrolyte, we are able to directly probe the effect of O_2_ saturation on OER rate. Fig. S2 (ESI†) shows EC-MS data for the oxidation of H_2_^18^O in (a) He-saturated and (b) ^16^O_2_-saturated ^18^O-labeled electrolyte. The presence of ^16^O_2_ makes no difference to the OER rate, measured *via* the ^18^O_2_ signal at *m*/*z* = 36. This is consistent with results by Marc Koper and coworkers, showing that the saturation of the electrolyte with O_2_ makes no difference to activity in alkaline OER.^57^

In order to further lower the potential limit for oxygen detection, Ru foam samples with a roughness factor of ∼3000 (see below) were synthesized on glassy carbon substrates by cathodic electrodeposition at −6 V_RHE_ of ruthenium from a solution of 10 mM RuCl_3_ under vigorous hydrogen evolution conditions.^50^ The synthesized Ru “foam” was porous as shown by the scanning electron microscope image in the inset of Fig. 2d. Using the porous Ru foam, oxygen was observed at potentials as low as 1.30 V_RHE_, only 70 mV above the standard thermodynamic potential for water oxidation, 1.23 V_RHE_, Fig. 2d (and possibly 1.29 V_RHE_, see Fig. S3, ESI†). Notably, the current response as a function of time in Fig. 2d shows a steady decay in the current over the entire potential hold, which can be attributed to the large capacitative charge for the high surface area foam samples, in contrast to the O_2_ signal, which rises only slightly during the potential hold. Thus the direct detection of oxygen at potentials lower than the observable onset of current can be reliably distinguished from charging current and enables the mechanistic investigation of oxygen evolution reaction kinetics closer to the equilibrium conditions.

The detection of oxygen at such low nominal overpotentials may seem surprising given density functional theory calculations that suggest an OER limiting potential of ∼1.43 V_RHE_^27,58,59^ (∼200 mV overpotential) on the optimal catalyst, following the linear scaling relationships between the *O, *OH and *OOH intermediates. Here, we experimentally show that this value does not correspond to the lowest measurable experimental onset potential. This is because, while the rate of the reaction decreases exponentially with applied potential, the exponential function never reaches zero. At low overpotentials, the reaction can still proceed, although at a slower rate than high potentials where all steps are predicted to be energetically favorable. Thus, the measurable onset of OER is only limited by the sensitivity of the oxygen detection system.

### Oxygen evolution activity on RuO_*x*_

**Fig. 3:**
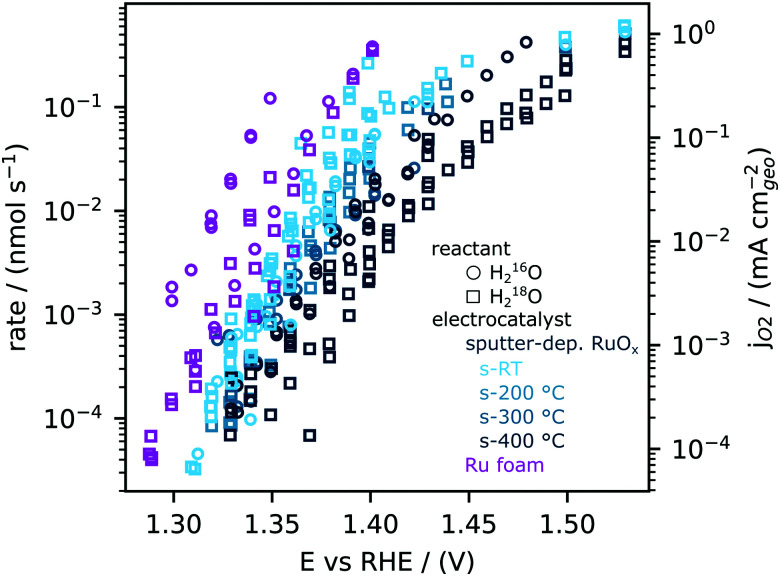
Oxygen production rates measured using EC-MS across a variety of RuO_*x*_ (films sputtered at room temperature, 200 °C, 300 °C and 400 °C) and Ru foam samples synthesized by ruthenium cathodic electrodeposition at −6 V_RHE_ from a solution of 10 mM RuCl_3_ under vigorous hydrogen evolution conditions. Activity is normalized to geometric area. Measurements were made in both unlabelled 0.1 M HClO_4_ (open circles) and labelled 0.1 M HClO_4_ in 97% H_2_^18^O (open squares) electrolyte.

We next quantitatively compare the oxygen flux measured as a function of potential for the sputtered RuO_*x*_ films and the high surface area Ru foam measured in labelled and unlabelled electrolyte, as shown in Fig. 3. The flux of oxygen generated at any given potential is about an order of magnitude higher on the room temperature sputtered films compared to those sputtered at 400 ^0^C. All samples were measured in a potential range that makes use of the ∼5 orders of magnitude dynamic range of the EC-MS setup (∼10^−5^ to ∼ 1 nmol s^−1^ cm^−2^). All of these measurements and more activity data are available in the database of the accompanying repository. The corresponding OER current density, obtained by converting the moles of oxygen to a current using Faraday's law of electrolysis is shown on the right *y* axis of Fig. 3. The current densities obtained per geometric surface area at 1.45 V_RHE_ for the sputtered films ranges from ∼0.1 mA cm^−2^_geo_ for the 400 °C sputtered film to ∼1 mA cm^−2^_geo_ for the room temperature sputtered film. These current densities are at least two orders of magnitude larger than that observed on single crystal RuO_2_(110), where ∼5 μA cm^−2^_geo_ was observed at similar potentials. Considering surface X-ray diffraction studies on single crystal RuO_2_(110) have demonstrated that the surfaces are atomically smooth with only the top monolayer contributing to OER,^21,22,60^ we hypothesize that the sputtered films have a larger electrochemically active surface area per geometric area (roughness factor) compared to single crystals.

**Fig. 4:**
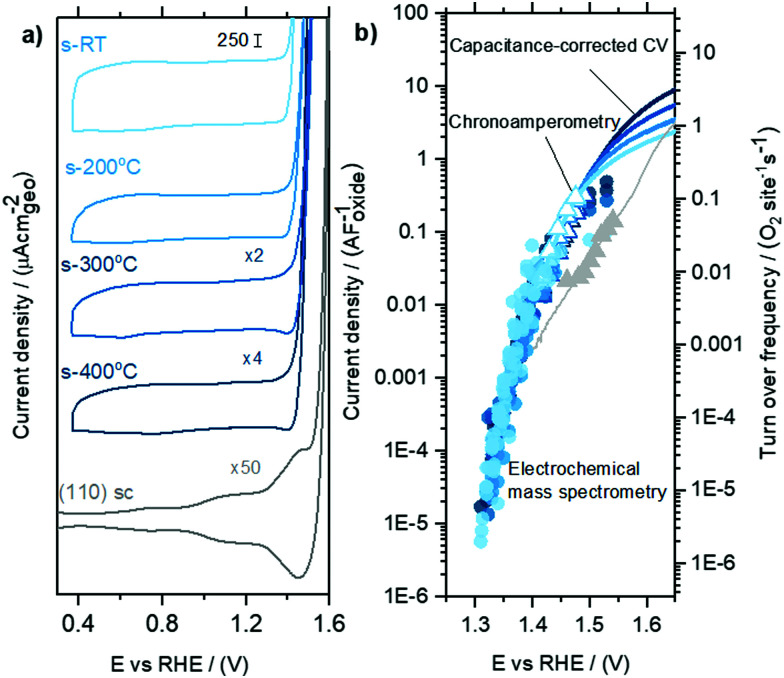
Comparing capacitance-normalized EC and EC-MS activity for sputter-deposited RuO_*x*_ films and RuO_2_(110) single crystal (a) cyclic voltammetry of RuO_*x*_ films sputtered at different temperatures, with single crystal data included as reference (b) capacitance-normalized partial OER current densities. Data points in circles are based on the oxygen measured in EC-MS, while triangles and lines are potentiostatic measurements and capacitance corrected linear sweep data measured in a rotating disk setup. The right *y*-axis shows the corresponding turn over frequency, calculated using the capacitance value of 115 μF cm^−2^_oxide_ and active site density value of 5 sites nm^−2^_oxide_ based on the RuO_2_(110) surface.

Cyclic voltammetry of sputter-deposited films between 0.4 V_RHE_ and 1.3 V_RHE_ show that the capacitance decreases from the room temperature sputtered samples to those sputtered at 200 °C, 300 °C and 400 °C, Fig. 4a and Fig. S4 (ESI†). There was no effect of O_2_ saturation of the electrolyte on the parts of the CV relevant to OER (Fig. S5, ESI†). Interestingly, the capacitance of sputter-deposited films, which could be estimated from normalizing the current density by the scanning rate, are greater than single crystals by approximately one order of magnitude. The specific capacitance normalized to electrochemically active surface area (ECSA) of amorphous RuO_*x*_ has been estimated to be a bit higher at 200 μF cm^−2^_ECSA_,^35,36^ but its measurement is challenging due to the difficulty of independently measuring the ECSA. In this work, we will use our value for the capacitance of RuO_2_(110), 115 μF cm^−2^_ECSA_ (Fig. S6, ESI†), to estimate roughness factors of the other films. The specific capacitance of 400 °C-sputter-deposited films is ∼15–20 times larger (2100 μF cm^−2^_geo_) than that of RuO_2_ single crystal surfaces, implying a roughness factor of 15–20. The specific capacitance of sputter deposited films decreases with increasing deposition temperature from room temperature, with ∼6 times greater capacitance (12 500 μF cm^−2^_geo_) for RuO_*x*_ deposited at room temperature compared to that sputter-deposited at 400 °C, implying a roughness factor of ∼100. The Ru foam's roughness factor of ∼3000 was calculated based on its specific capacitance of ∼400 000 μF cm^−2^_geo_ (see Fig. S3, ESI†). We attribute this difference in capacitance to the larger density of accessible sites in the films deposited at lower temperature, possibly owing to the formation of less compact films.

The geometric OER activity of sputter deposited films is 2–3 orders of magnitude higher (∼20 mA cm^−2^_geo_ at 1.53 V_RHE_ for room temperature sputter deposited RuO_2_) than that of the RuO_2_(110) (∼0.015 mA cm^−2^_geo_ at 1.53 V_RHE_), measured from CV and potentiostatic measurements. This difference is partly explained by electrochemically active surface area (ECSA) as measured by capacitance. Notably, however, the activity of the sputter deposited RuO_*x*_ films normalized to the capacitance is still ∼5 times higher than the RuO_2_(110) single crystal surface (Fig. 4b). While the OER activity normalized to geometric surface area for the sputter deposited films decrease with increasing sputtering temperature, the activity normalized to the average capacitance found between 0.4 V_RHE_ and 1.3 V_RHE_, and consequently the turnover frequency, are similar for the sputtered films and ∼1 order of magnitude higher than RuO_2_(110), as shown in Fig. 4b. Other single-crystal surfaces RuO_2_(100) and (101) have activities only slightly higher than RuO_2_ (110),^22,61,62^ and so the sputter-deposited films have significantly higher ECSA-normalized activity than any single crystal facet. This observation suggests that the differences in activity between the single crystal surfaces and the sputter deposited films is not only a result of differences in accessibility of active sites alone, but also possibly from the intrinsic activity of these sites for OER. Differences in the activity between single crystal and sputter deposited films can originate from different coordination environments of the active Ru sites, which has been shown to significantly impact binding energy of reaction intermediates and OER activity.^22,61,62^ Recent experimental data on well-defined single crystals of RuO_2_ (110), (100) and (101) surfaces demonstrates that a change in the coordination environment of oxygen atoms surrounding the active Ru site and the predicted Ru–O–Ru bond angle can result in a weakening in binding energy between the most active RuO_2_(100) surface and the thermodynamically stable RuO_2_(110) surface of ∼0.04 eV^22^ and consequently an increase in OER activity of ∼5 times per active Ru site, based on a Tafel slope of 60 mV decade^−1^.^22^ Theoretical work by Nørskov and coworkers. considered a diverse range of active Ru sites on kinked RuO_2_(121) surfaces^61,62^ and showed that the oxygen binding energetics, Δ*G*_O_ − Δ*G*_OH_ on different surface sites can vary by ∼0.7 eV. In summary, while these sputter deposited oxides can produce larger OER activity compared to single crystal surfaces at a given potential, their OER mechanism at low potential is not understood.

### Tafel slope at low overpotential

By co-plotting the OER partial current density determined from the oxygen flux along with the electrochemical activity measurements, we find that all the four sputter deposited films exhibit a new Tafel regime with a slope of ∼25 mV decade^−1^ at low potentials, which has not been previously experimentally detected on Ru-based oxides. Three distinct Tafel regimes have been observed on all the sputter deposited films (Fig. 4b and Fig. S7, ESI†). First, in the low overpotential regime (<1.40 V_RHE_) the newly revealed Tafel slope is ∼25 mV decade^−1^. Second, the next regime has a Tafel slope of 60 mV decade^−1^ and lasts till ∼1.47 V_RHE_ on the room temperature sputter deposited film, and till ∼1.52 V_RHE_ on the high temperature sputter deposited film. A Tafel slope of ∼60 mV decade^−1^ has also been found in previous work on oriented RuO_2_ single crystals,^21,22,45^ epitaxial thin films^44,63,64^ and nanoparticles^19^ at potentials greater than 1.50 V_RHE_. Finally, at potentials above ∼1.55 V_RHE_ and ∼1.60 V_RHE_ for the room temperature and 400 °C sample respectively, the Tafel slope increases to ∼120 mV decade^−1^.

Potential-dependent Tafel slope can be attributed to one or more of several reasons. First, changes in the surface coverage of intermediates as a function of potential^22,42,65^ can result in different Tafel slopes across a range of potential for the same rate determining step as elucidated by the microkinetic analysis in the seminal work of Bockris^46,65^ and more recently by Shinagawa *et al.*^42^ and Mefford *et al.*^41^ Second, changes in the Tafel slope can be attributed to a change in the reaction mechanism with potential. Third, the superposition of different sites contributing to the overall activity could also result in different Tafel slopes, depending on which site dominates the activity at a given potential. From the Butler Volmer equation:1
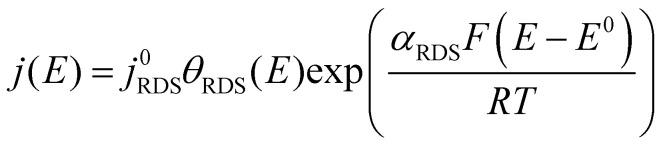
where *E* is the potential, *E*^0^ is the standard thermodynamic potential (1.23 V_RHE_), *R* is the gas constant, *T* is the temperature, *α*_RDS_ is the symmetry factor of the rate determining step (RDS), *θ*_RDS_ is the potential-dependent coverage of the active state (*i.e.*, the state from which the RDS takes place), and *j*^0^_RDS_ is the theoretical exchange current density of the active state, which is the activity at *E*^0^ if there were full coverage of the active state at this potential. If we assume that *θ*_RDS_ is independent of potential, the Tafel slope is given by:2
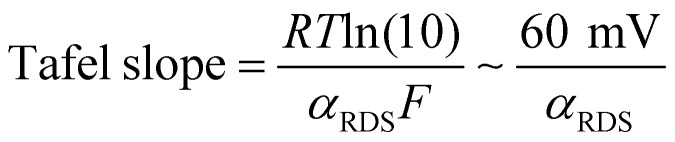


This cannot explain a Tafel slope of 25 mV decade^−1^, considering the value of α_RDS_ can only be between 0 and 1. This is the case even if the mechanism changes as a function of potential, or multiple active sites with different *j*^0^_RDS_ and *α*_RDS_ contribute. In the latter case, furthermore, the Tafel slope should decrease at higher potential as the site with the larger *α*_RDS_ increasingly dominates the current, opposite to the observed behavior. Therefore, we conclude that *θ*_RDS_ has a potential dependence.

Based on the changes in the Tafel slope, we can also rule out a purely chemical rate-determining step. Recent measurements of X-ray adsorption and discharge current on IrO_*x*_ indicated that the rate-determining step may not be directly coupled to an electron transfer.^66^ In this case, the potential dependence comes only from the coverage of the active state, *θ*_RDS_, which would eventually reach 1 at high overpotentials, resulting in an infinitely large Tafel slope. Instead, on the sputtered films, we observe a Tafel slope approaching 120 mV s^−1^ with increasing overpotential as expected with *α*_RDS_ = 1/2. A chemical nature of the rate-determining step would imply a steep rise in the Tafel slope once a surface coverage of 1 is reached. We attempted to fit the results with both modifications to eqn (1) but they resulted in worse fits with much higher mean square errors.

Here, we propose that the changing Tafel slope comes from the potential dependence of the coverage of surface species that can participate in the rate-determining step (SI under “Kinetic model”). From the microkinetic rate equation, one can incorporate this coverage dependence in an “effective transfer coefficient”,^42,46^α*. The effective transfer coefficient, unlike the symmetry factor of the rate-determining step, is not restricted to be between 0 and 1. As demonstrated in the ESI† (eqn (S39), ESI†), the effect of surface coverage on the effective transfer coefficient can be explained by:3
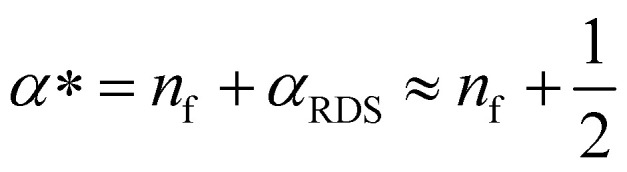
where *n*_f_ is the number of electrons that need to be transferred per active site to reach the active state (from which the RDS occurs) from the actual state of the surface and *α*_RDS_ is the symmetry factor of the RDS (for which we assume the usual value^67^ of 1/2, in agreement with the Tafel slope of 120 mV decade^−1^ that we observe at high overpotentials).

**Fig. 5:**
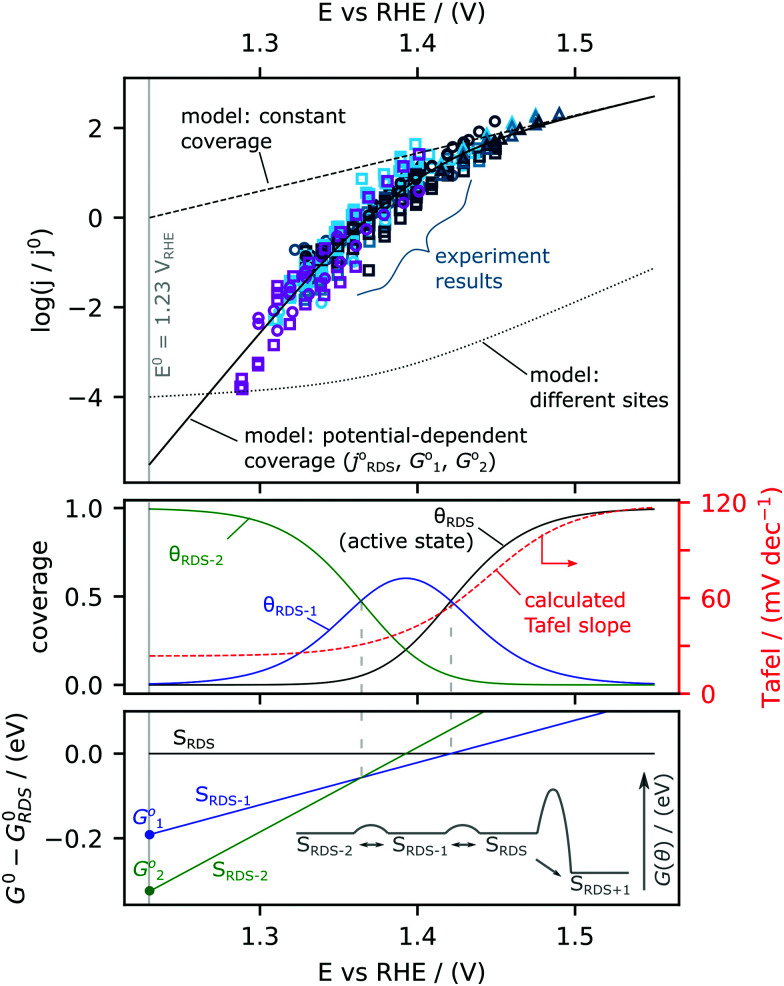
Microkinetic model to simulate changes in Tafel slope. (Top) Experimental activity measurements (open symbols) compared to three models with (1) no coverage change (dashed) (2) presence of two different active sites (dotted) and (3) Changing coverage of the surface species as a function of potential (solid). (Middle) The coverages and (bottom) the Gibbs Free Energy of the three different states achieving the best-fit to the experimental data are shown. The surface state corresponding to the rate-determining step (RDS) is denoted as S_RDS_, the states preceding the RDS are denoted as S_RDS-1_ and S_RDS-2_. Inset shows a diagrammatic description of the energetics of the different surface states.

The Tafel slope at the lowest nominal overpotentials measured in this study was about 25 mV decade^−1^, implying that at these potentials, α* = 2.5 (by eqn (2) with α* replacing α_RDS_) and *n*_f_ = 2 (by eqn (3)). In other words, our data indicate the equilibrium coverage of intermediates at low potentials is such that there needs to be two electron transfers per site before reaching the RDS. Therefore, we model the reaction assuming three distinct surface termination states, denoted S_RDS_, S_RDS-1_ and S_RDS-2_, where S_RDS_ is the active state from which the rate-determining step takes place, and S_RDS-1_ and S_RDS-2_ precede it by one and two electron transfers, respectively. We assume that S_RDS_, S_RDS-1_ and S_RDS-2_ are in equilibrium. Consequently, at any given potential, the surface coverage of these states *θ*_RDS_, *θ*_RDS-1_ and *θ*_RDS-2_ can be obtained based on the relative standard free energies of these three states at a given potential, shown in Fig. 5. For mathematical details of the model, see the ESI.†

We do not consider the back reaction, the oxygen reduction reaction (ORR) in this model. Equilibrium concepts are discussed in detail in the ESI.† Briefly, in the potential range spanned by our data (*E* > 1.29 V_RHE_), we predict that any ORR current would be at least 5 orders of magnitude lower than the OER current, and thus negligible, as illustrated in Fig. S8 of the ESI.† An insignificant ORR contribution is consistent with the lack of any effect of O_2_ partial pressure on the measured OER rate (Fig. S2 and S5, ESI†) and by the fact that the scatter in the normalized activity data increases if plotted against Nernst-adjusted overpotential rather than against potential on the RHE scale (Fig. S8a and b, ESI†). Note that with ORR left out of the model, the concept of overall equilibrium does not come into play, and *E*^0^ = 1.23 V_RHE_ serves only as a reference potential.

The physical nature of *S*_RDS_, *S*_RDS-1_ and S_RDS-2_ can be hypothesized based on DFT calculations and *in situ* surface diffraction measurements in previous works^21,22^ on single crystal RuO_2_(110) surface, where it has been proposed that the rate-determining step is the final deprotonation step, *OO—H → O_2_ + (H^+^ + e^−^) + *. In this case, the RDS-active state (*S*_RDS_) corresponds to active coordinatively unsaturated sites (CUS) filled with *OO—H, *S*_RDS-1_ corresponds to all CUS sites filled with *O since one electron transfer is required to reach the RDS (*O + H_2_O → *OO –H + (H^+^ + e^−^), Δ*G*^0^_1_) and S_RDS-2_ corresponds to all CUS sites filled with *OH since two electron transfers are required to reach the RDS (*OH + H_2_O → *OO—H + 2(H^+^ + e^−^), Δ*G*^0^_2_). We reproduce the experimentally observed changes in Tafel slope by fitting the expanded version of Eqn 1 (eqn (S44) of the ESI†) with three parameters: *j*^0^_RDS_, Δ*G*^0^_1_ and Δ*G*^0^_2_ and find that the best fit is given by *j*^0^_RDS_ = 0.9 mA F^−1^ (TOF^0^ = 3 × 10^−4^ s^−1^), Δ*G*^0^_1_ = −0.19 eV, and Δ*G*^0^_2_ = −0.33 eV. Details of fitting procedure are in ESI.† The result of the fitting is shown in Fig. 5. In the top panel, the model current density is co-plotted with the experimental data. The modeled coverages are shown in the middle panel, and the modeled standard free energies of the states relative to the active state are plotted in the lower panel. The redox transition from S_RDS-2_ to S_RDS-1_ occurs at 1.35 V_RHE_ and the redox transition from S_RDS-1_ to S_RDS_ occurs at 1.41 V_RHE_. These values are in fairly good agreement with the theoretically constructed Pourbaix diagram constructed for the ideal surface of RuO_2_(110)^21^ where the transition from *OH to *O species (*OH → *O + (H^+^ + e^−^)) occurs at 1.22 V_DFT-RHE_ and the subsequent transition from *O to *OOH species (*O + H_2_O → *OO—H − (H^+^ + e^−^)) occurs at 1.42 V_DFT-RHE_. The discrepancies between the numerical values can be attributed to differences in the binding energetics of oxygenated intermediates on the sputter-deposited films relative to the CUS sites on the (110) surface. Notably, based on this model, at low overpotential, the rate of the reaction is strongly dependent on the coverage of species that can participate in the RDS. Therefore, at potentials <1.35 V_RHE_, a factor-10 improvement in activity would require stabilizing the RDS state with respect to the state dominating the surface (S_RDS-2_) by only 30 meV, which results in an increase in density of species that can be involved in the RDS. In contrast, if the energetics of *S*_RDS_ and *S*_RDS-2_ were unchanged, the activation barrier for the rate-determining step would have to be reduced by 120 meV to achieve the same improvement (see ESI† for details: eqn (S45), ESI†). These results thus highlight the importance of optimising the energetics of pre-OER surface species to improve kinetics in the low-overpotential regime.

The identification of a new Tafel regime at potentials lower than 1.40 V_RHE_ calls into question the determination of exchange current density for oxygen evolution reaction. The exchange current is defined as the magnitude of the current density of the forward reaction at zero overpotential and serves as a metric to compare the activity of different catalysts. The value is typically extracted by extrapolating the kinetics observed at potentials >∼1.50 V_RHE_ to the equilibrium potential, assuming a constant Tafel slope. Based on our results, extrapolating data from ∼1.5 V_RHE_, results in an exchange current density of 0.9 mA F^−1^ (TOF of 3 × 10^−4^ s^−1^). However if we extrapolate instead from the low-overpotential regime <∼1.4 V_RHE_, the exchange current density is six orders of magnitude lower, ∼1 nA F^−1^ (TOF ∼ 3 × 10^−10^ s^−1^). This corresponds to a value of ∼2 × 10^−10^ mA cm^−2^_ECSA_, or ∼10^−8^ mA cm^−2^_geo_ for s-25 °C RuO_*x*_, which has a roughness factor of ∼50. The ECSA-normalized exchange current density for OER on RuO_2_ is thus about 10 orders of magnitude lower than the exchange current density obtained for hydrogen evolution on low-index single crystal Pt surfaces, ∼1 mA cm^−2^_ECSA_.^68^ Clearly, both the experimental onset potential and the exchange current density are ambiguous metrics for OER activity, with an accuracy depending on the measurement sensitivity for oxygen. Therefore, we recommend using turn-over frequency for O_2_ production at a given nominal overpotential as the best fundamental metric whenever it is possible to estimate the number of active sites.

## Conclusions

In this work, we used highly sensitive mass spectrometric O_2_ detection to measure the OER activity of RuO_*x*_ at low overpotentials. Direct detection of O_2_ at low overpotentials is necessary because the electrocatalytic current can be small compared to other currents such as the capacitive charging current. Using ^18^O isotopic labeling to improve sensitivity, we detect oxygen from 1.32 V *vs.* RHE on sputter deposited amorphous RuO_*x*_ and 1.30 V *vs.* RHE on Ru foam, nominal overpotentials of only 90 mV and 70 mV, respectively. To our knowledge, this sets the record for the lowest overpotential at which electrocatalytic O_2_ evolution has been measured. Complementing the dynamic range of mass spectrometry by synthesizing samples with a wide range of roughness factors, we obtained quantitative data spanning more than six orders of magnitude, revealing a new Tafel regime of 25 mV decade^−1^ below ∼1.4 V_RHE_. We attribute this strong potential dependence to a potential-dependent coverage of the surface state active in the rate-determining step, in line with recent kinetic and spectroscopic insight. Using these ultrasensitive activity measurements as inputs to a mechanistic model, we provide evidence for an electron transfer coupled rate-determining step from an active surface state in equilibrium with two other surface states more stable by energies of −0.19 eV and −0.32 eV at 1.23 V_RHE_. In addition to challenging the conventional notion of onset potential and exchange current density in water oxidation electrocatalysis, this data and technique opens a new window through which to probe the energetics of electrocatalytic intermediates and inform rational catalyst design of better and more scalable acid–electrolyte oxygen evolution catalysts.

## Experimental methods

### Sample preparation and characterization

RuO_*x*_ films were prepared by reactive sputtering on glassy carbon substrates. The substrates (0.196 cm^2^) were polished before the deposition process with 0.25 μm diamond paste. A thin layer (∼5 nm) of Ti was first sputter deposited and pressure of 10 mTorr from a Ti target to facilitate adhesion of the RuO_*x*_ on the substrate. RuO_*x*_ films were then deposited from a metallic Ru target at the noted temperature in a pressure of 3 mTorr with an Ar:O_2_ flow ratio of 5 : 2. A 50 W power source was used. The target for sputtering was purchased from Kurt J. Lesker. For isotopically labelled oxides, the oxygen gas in the chamber was ^18^O_2_ (manufacturer) with a purity of 99%.

The Ru foam sample was synthesized by cathodic electrodeposition using a glassy carbon substrate. The deposition potential used was −6 V_RHE_ (in the potential region for vigorous hydrogen generation) and deposition solution was 10 mM RuCl_3_. The deposition time was 2500 seconds. X-ray diffraction spectra were collected using a PANanalytical X’pert PRO machine with an X-ray wavelength of 1.54 Å for the Cu_Kα_ line. AFM images collected using a Veeco Nanoscope IV. The XRD data collected was normalized to the noise level.

### Electrochemical measurements

Electrochemical measurements were performed using a Biologic SP-300 potentiostat in a four-neck glass cell with a three-electrode configuration. ∼150 mL of 0.1 M HClO_4_ (70% Veritas® double distilled) was prepared using deionized water (Millipore, >18.2 MΩcm). A 4 M saturated Ag/AgCl reference electrode was used, which was calibrated to the RHE scale in the 0.1 M HClO_4_ solution. A large Pt wire was used as the counter electrode. The pH of the solution was measured using a pH meter. The electrolyte was bubbled with Ar (for cyclic voltammetry measurements) or O_2_ (for activity measurements). Electrical impedance spectroscopy was performed under open circuit with an amplitude of 10 mV. The high frequency intercept of the real resistance obtained from the Nyquist plot was used as the solution resistance.

### EC-MS

Electrochemistry-mass spectrometry measurements were performed using an interface system purchased from Spectro Inlets A/S. The setup is described in reference.^47^ An internal volume fabricated into a silicon-on-insulator microchip functions as a microscopic headspace which equilibrates with the working volume and delivers electrochemical products to the vacuum chamber without differential pumping. The mass spectrometer was a QMG electronics system and QMA 125 mass analyzer from Pfeiffer Vacuum, and the potentiostat was a Biologic SP 150. The electrolyte was 0.1 M HClO_4_ in either deionized water (Millipore) or 97% H_2_^18^O isotope-labelled water (Medical Isotopes). The working electrode was connected through a 100 Ohm resistor to stabilize the potential, and all reported potentials are corrected by subtracting the current times this resistance. (This makes a negligible difference at the lowest over-potentials of interest, where the current is on the order of a microampere.)

### Data and modelling

#### Data analysis

All data was analyzed and organized with the open-source “*In situ* Experimental Data Tool” (ixdat, https://ixdat.readthedocs.org) and a homemade database, all available at https://github.com/ixdat/LowOverpotentialRegime. Additional data sets were obtained on each sample to verify reproducibility and are shown in Fig. S9–S16 (ESI†).

Fitting was done by minimizing the mean square error on the logarithm of the modeled current density, with each activity point measured by potential hold in EC-MS (<1.45 V_RHE_) or conventional RDE electrochemistry (>1.4 V_RHE_).

## Conflicts of interest

The authors do not have any conflicts of interest to declare.

## Supplementary Material

EE-015-D1EE03914H-s001
