# Supplementary material for: The low overpotential regime of acidic water oxidation part I: the importance of O2 detection
Source: Energy Environ Sci. 2022 Mar 19;15(5):1977–87. doi: 10.1039/d1ee03914h (PMC9116083; doi:10.1039/d1ee03914h)
Supplement: EE-015-D1EE03914H-s001 [file EE-015-D1EE03914H-s001.pdf]

Electronic Supplementary Material (ESI) for Energy & Environmental Science.
This journal is © The Royal Society of Chemistry 2022

Supplementary Information for: The Low-Overpotential Regime of Water Oxidation in Acid - Part I: The Importance of O2 detection

Soren B. Scott[1], Reshma R. Rao[2], Choongman Moon[1], Jakob E. Sørensen[1], Jakob Kibsgaard[1], Yang Shao-Horn[2], and Ib Chorkendorff[1]

[1]SurfCat Section for Surface Physics and Catalysis, Department of Physics, Technical University of Denmark, Kgs. Lyngby, Denmark

[2]Department of Mechanical Engineering, Massachusetts Institute of Technology, Cambridge, Massachusetts, USA

March 24, 2022

Contents

1 Characterization of samples **2**

2 OER in O2-saturated electrolyte **3**
- 2.1 O_2 partial pressure at the electrode surface . . . 3
- 2.2 Oxidataion of $H_2{}^{18}O$ in the presence of $^{16}O_2$ . . . 3
- 2.3 Mechanistic implications . . . 5

3 Pushing the limit with Ru foam **6**

4 Cyclic voltammotry **7**

5 Tafel regimes on sputter-deposited Ru films **9**

6 Kinetic model **10**
- 6.1 Static coverage models . . . 11
- 6.2 Potential-dependent coverage . . . 12
- 6.3 Relative importance of coverage and driving force . . . 15
- 6.4 Fitting procedure for the coverage dependent model . . . 16
- 6.5 Remarks on equilibrium . . . 17

7 Database contents **20**

1 Characterization of samples

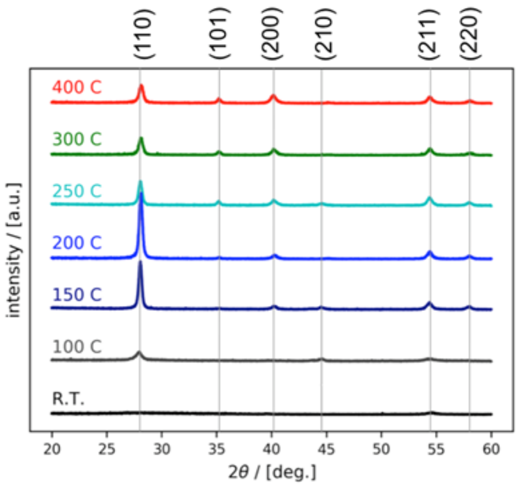

Figure 1: X-Ray diffraction of RuOx sputtered films with a sputtering temperature of 400°C (red), 300°C (green), 250°C (light blue), 200°C (blue), 150°C (dark blue), 100°C (grey) and room temperature (black). The peaks corresponding to rutile RuO2 have been labeled. The spectra were taken with a grazing-incidence (alpha-2θ) geometry with an incidence angle of 0.5°.

Deposition Temperature	RMS Roughness
Room Temp	1.416
100°C	1.609
150°C	1.782
200°C	1.544
250°C	1.961
300°C	1.958
400°C	2.34

Table 1: Root mean square roughness values for RuOx films deposited at temperatures from 400°C to room temperature. The films are nominally smooth with a root mean square roughness ranging from 1-3 nm.

2 OER in O2-saturated electrolyte

2.1 O_2 partial pressure at the electrode surface

The EC-MS activity measurements presented in the main text were performed with the electrolyte saturated with helium, chosen as an inert gas without interfering mass spectrometry signals. The measurements were thus *not* done under the standard condition of saturation with 1 bar O_2, the condition for which the equilibrium potential is 1.23 V_{RHE}. This is why we refer to 1.23 V as the *standard* equilibrium potential and the difference between an applied potential and 1.23 V_{RHE} as the *nominal* overpotential.

The lack of O_2 saturation influences the OER equilibrium potential according to the Nernst equation:

$$U_{\mathrm{eq}} = U^{\circ} + \frac{RT}{4\mathcal{F}} \ln(a^{\mathrm{O_2}}) \,, \tag{1}$$

where U^{eq} is the equilibrium potential, U° is the *standard* equilibrium potential, R is the gas constant, T is the temperature, $\mathcal{F}$ is Faraday's constant, and $a^{\mathrm{O_2}}$ is the O_2 activity. The O_2 activity is defined as the ratio of the actual O_2 concentration to that of a solution saturated with O_2:

$$a^{\mathrm{O_2}} = \frac{c_{\mathrm{el}}^{\mathrm{O_2}}}{c_{\mathrm{sat}}^{\mathrm{O_2}}} = \frac{c_{\mathrm{el}}^{\mathrm{O_2}}}{1\,\mathrm{bar}/K_{\mathrm{H}}^{\mathrm{O_2}}} = \frac{c_{\mathrm{el}}^{\mathrm{O_2}}}{1.3\,\mathrm{mM}} \tag{2}$$

Where $c_{\mathrm{el}}^{\mathrm{O_2}}$ is the O_2 concentration at the electrode and we used the Henry's Law constant of O_2 in water, $K_{\mathrm{H}}^{\mathrm{O_2}}$, to calculate the saturation concentration, $c_{\mathrm{sat}}^{\mathrm{O_2}}$.

For a light gas like O_2, the transport of oxygen away from the electrode surface is limited by diffusion through the working volume[1], and so the concentration at the electrode surface, can be estimated using Fick's first law of diffusion:

$$c_{\mathrm{el}}^{\mathrm{O_2}} = \frac{\dot{n}^{\mathrm{O_2}}}{A_{\mathrm{el}}} \frac{L}{D^{\mathrm{O_2}}} \,, \tag{3}$$

where $\frac{\dot{n}^{\mathrm{O_2}}}{A_{\mathrm{el}}}$ is the flux density of oxygen produced at the electrode, L is the working distance between the electrode and the chip membrane, and $D^{\mathrm{O_2}}$ is the diffusion constant of O_2 in water. The largest deviation from standard conditions occurs at the smallest concentration of O_2, and thus at the smallest O_2 production rates, which in these experiments means a flux density at our detection limit of around 1 pmol/s/cm^2.

Plugging in $\frac{\dot{n}^{\mathrm{O_2}}}{A_{\mathrm{el}}} = 1$ pmol/s/cm^2 gives, moving up through the equations: $c_{\mathrm{el}}^{\mathrm{O_2}} = 5 \cdot 10^{-11}$ M, $a^{\mathrm{O_2}} = 4 \cdot 10^{-11}$, and $U_{\mathrm{eq}} = 1.12$ V_{RHE}.

Thus the equilibrium is more cathodic by about 110 mV in the most extreme case given the actual concentration of O_2 on the surface. However, as we discuss in more detail in Section 6.5 of this SI, the OER is kinetically driven, so it remains for now an open question of whether the shift in equilibrium potential influenes the *rate* of OER. At high overpotentials, this can be tested simply, by measuring the OER current with and without O_2 in the electrolyte, as shown in this SI in Figure 5.

It is more challenging to determine whether the presence of O_2 effects the OER in the low overpotential regime where detection of O_2 is necessary for reliable OER activity measurement. To overcome this challenge, we designed an experiment in which we use isotope labeling and the gas-switching capability of chip EC-MS.

2.2 Oxidataion of $H_2^{18}O$ in the presence of $^{16}O_2$

Figure 2a shows an EC-MS experiment testing the effect of O_2 saturation on OER. Starting from the left, the electrode is in He-saturated $H_2^{18}O$ electrolyte, and the potential is cycled. The oxygen produced is primarily $^{18}O_2$, but with about 10% $^{16}O^{18}O$ due to the isotopic impurity of the electrolyte (see ref. [2] for a note on isotopic distribution in OER). An activity measurement is made by stepping the potential to progressively higher working potentials in the range 1.33 V_{RHE} to 1.45 V_{RHE} with two-minute holds interspaced by resting periods at 1.23 V_{RHE}.

At about 3500 s in the measurement, the carrier gas in the chip is switched from He to isotopically natural O_2, which is 99.6% $^{18}O_2$. This increases the background level of $^{16}O^{18}O$ due to the small natural presence of ^{18}O, but has little effect on the $^{18}O_2$ signal.

The activity measurement is repeated in $^{16}O_2$-saturated electrolyte. The OER product, $^{18}O_2$, is clearly differentiable from the $^{16}O_2$ saturating the electrolyte. The data is calibrated by assuming 100% OER Faradaic efficiency on an IrO_2 electrode measured under identical conditions.

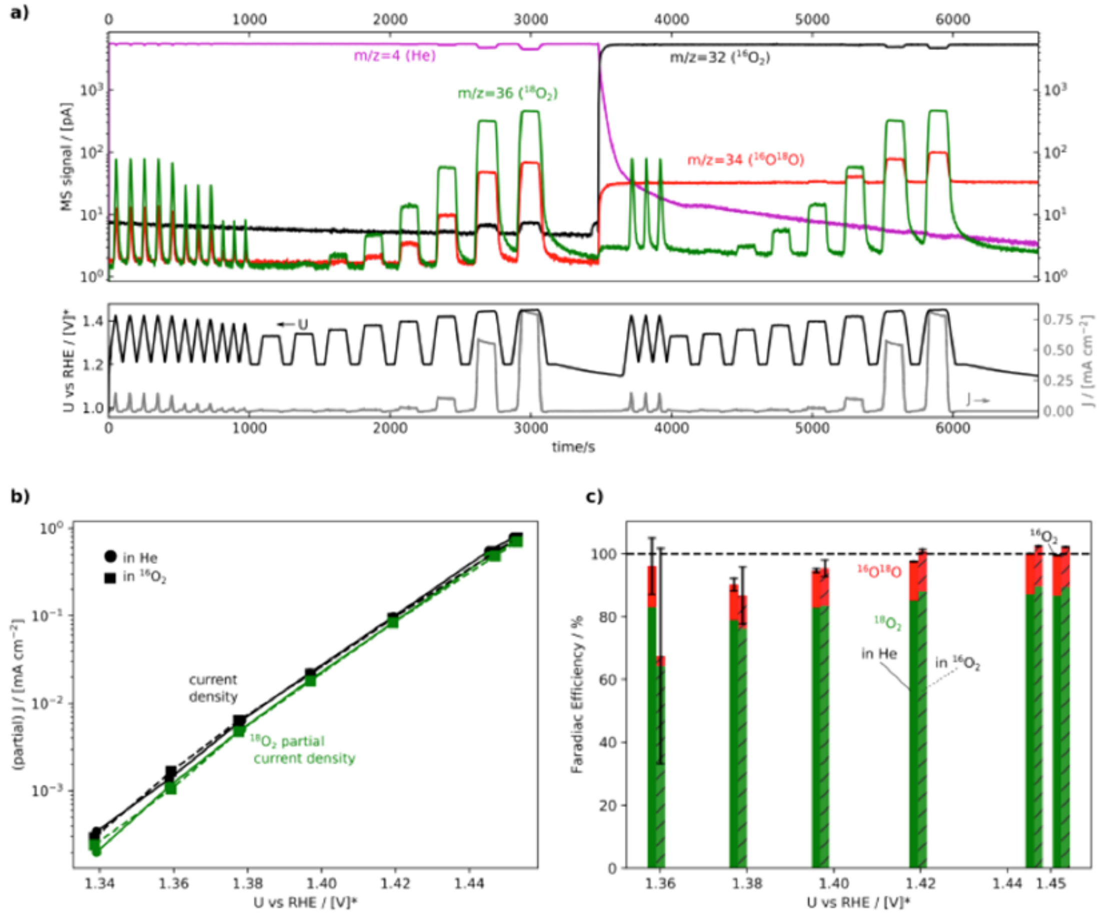

Figure 2: Activity of crystalline RuOx sputtered at 400°C in He-saturated and 16O2-saturated 0.1 M $HClO_4$ in $H_2{}^{18}O_2$. (a) Experiment as an EC-MS plot. (b) The current density (black) and the partial current density for $^{18}O_2$ (green) at the end of each constant-potential step. (c) The Faridaic eciencies for 18O2 (green) and $^{16}O^{18}O$ (red) as a function of potential. The error bar represents the uncertainty due to the standard deviation of the baseline m/z=34 MS signal.

Figure 2b shows the results af the activity measurement: Total current density and $^{18}O_2$ partial current density, calculated as $j^{O_2} = \dot{n}^{O_2}/(4\mathcal{F})$, are plotted vs the hold potential. This is done for both the measurement in He-saturated electrolyte (solid lines, circles) and in O_2-saturated electrolyte (dotted lines, squares). There is no discernable difference, i.e. the activity and $^{18}O_2$ partial current density are identical for OER in the two sets of conditions. The $^{18}O_2$ partial current density falls separates a bit from the total current density at the lowest overpotentials, where capacitive charging current is a significant fraction of the total, as described in the main text.

Figure 2c shows the Faradaic efficiency to $^{18}O_2$ and $^{16}O^{18}O$ as a function of potential in the two conditions (solid bars for He-saturated, striped bars for O_2-saturated). An error bar is put on the $^{16}O^{18}O$ signal, calculated by the standard deviation in the background signal divided by the current density. This is higher in O_2-saturated electrolyte, where the $^{16}O^{18}O$ background is higher. Allowing for these error bars, there is no measurable change in Faradaic efficiency for the isotopic O_2 products as a result of $^{16}O_2$-saturate of the electrolyte. This has an interesting mechanistic implications.

2.3 Mechanistic implications

The OER can be divided into four electron transfer steps, conventionally written as:

$$A. \qquad * + H_2O \longrightarrow {}^{*}OH + (H^+ + e^-) \tag{4}$$

$$B. \qquad {}^{*}OH \longrightarrow {}^{*}O + (H^+ + e^-) \tag{5}$$

$$C. \qquad {}^{*}O + H_2O \longrightarrow {}^{*}OOH + (H^+ + e^-) \tag{6}$$

$$D. \qquad {}^{*}OOH \longrightarrow O_2 + * + (H^+ + e^-) \tag{7}$$

As we will discuss in Section 6.2 of these SI, one of these steps can be considered the rate-determining step (RDS), and fitting of the results implies that there are two pre-catalytic states in equilibrium with the active state of the surface, from which the RDS takes place. If we assume that the pre-catalytic states are OER intermediates appearing in the above mechanistic scheme ($*$, ${}^{*}OH$, ${}^{*}O$, and ${}^{*}OOH$), then we can write the possible pre-catalytic equilibria as follows:

$$A \text{ as RDS.} \qquad {}^{*}O + H_2O \longleftrightarrow {}^{*}OOH + (H^+ + e^-) \longleftrightarrow * + O_2 + 2\,(H^+ + e^-) \tag{8}$$

$$B \text{ as RDS.} \qquad {}^{*}OOH + H_2O \longleftrightarrow O_2 + * + (H^+ + e^-) + H_2O \longleftrightarrow {}^{*}OH + O_2 + 2\,(H^+ + e^-) \tag{9}$$

$$C \text{ as RDS.} \qquad * + H_2O \longleftrightarrow {}^{*}OH + (H^+ + e^-) \longleftrightarrow {}^{*}O + 2\,(H^+ + e^-) \tag{10}$$

$$D \text{ as RDS.} \qquad {}^{*}OH + H_2O \longleftrightarrow {}^{*}O + H_2O + (H^+ + e^-) \longleftrightarrow {}^{*}OOH + 2\,(H^+ + e^-) \tag{11}$$

If A or B were the RDS, we would expect an euilibrium to exist involving O_2, and thus for the partial pressure of O_2 to effect the pre-catalytic equilibrium giving rise to the active state. This is discussed further in Section 6.5

If A were the RDS, we would further expect an isotope scrambling effect as the equilibrium would open up for the following equilibrium

$$* + {}^{16}O_2 + H_2^{18}O + 2\,(H^+ + e^-) \longleftrightarrow {}^{*16}O + H_2^{18}O + H_2^{16}O \longleftrightarrow * + {}^{16}O^{18}O + H_2^{16}O \tag{12}$$

As the ratio of ${}^{16}O^{18}O$ to ${}^{18}O_2$ produced by OER is the same in He-saturated and ${}^{16}O_2$-saturated electrolyte, this is not the case, we can rule out A as the rate-determining step.

If we instead assume that all steps except for the RDS are in equilibrium, then B as RDS would also put O_2 dissociation on the surface in a state of equilibrium, allowing us likewise to rule it out as RDS.

3 Pushing the limit with Ru foam

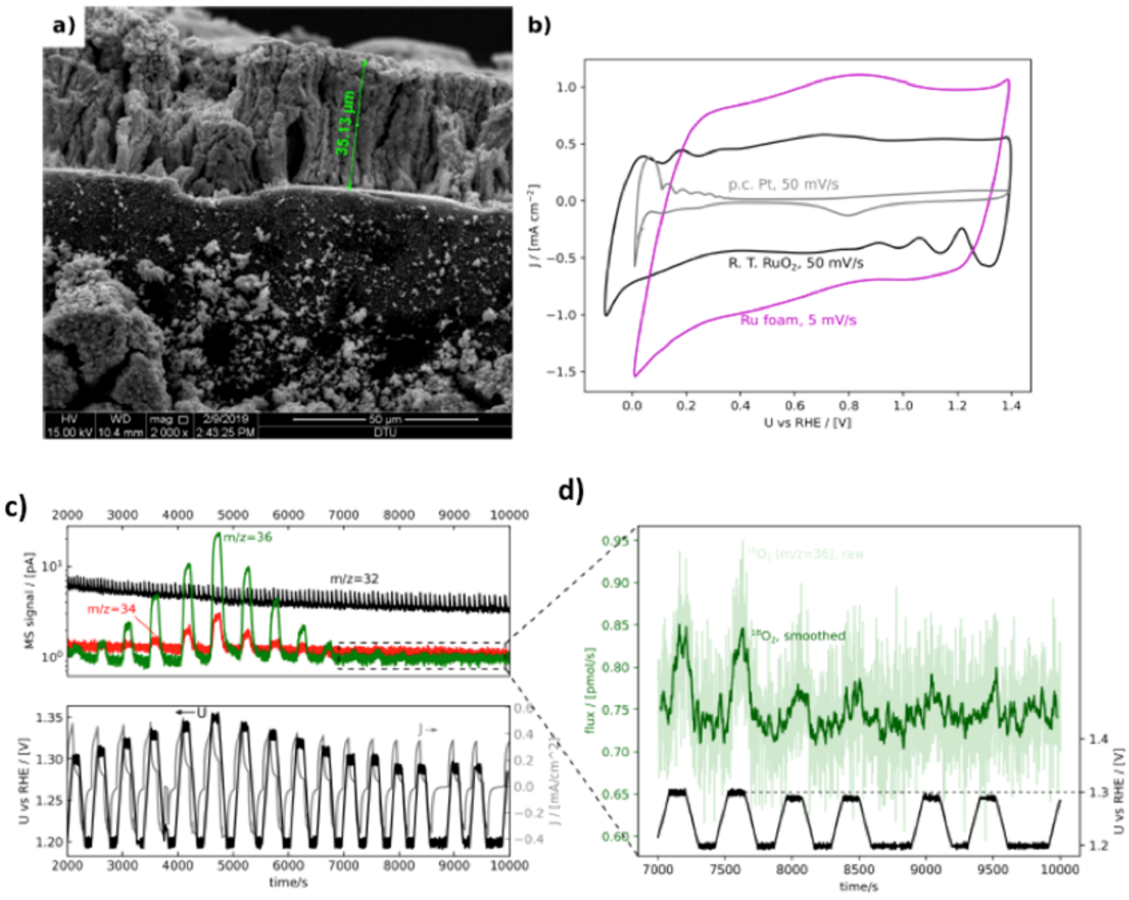

Figure 3: SEM image of Ru foam. (b) Cyclic voltammatry in 0.1 M HClO4 of a polycrystalline Pt electrode (grey), a room-temperature sputtered RuOx (black), and Ru foam (magenta). Note the different scan rates. The features just after the cathodic and anodic turns on the R.T. RuOx cyclic voltammogram are artifacts of the electrode arrangement in the EC-MS setup. (c) EC-MS plot with raw MS data from an activity measurement at low overpotentials on the Ru foam in O-18 labeled 0.1 M HClO4. (d) Zoom-in on the lowest overpotentials, with the calibrated 18O2 (m/z=36) signal (faint green). The solid green trace is a 15-point moving-average smoothing of the 18O2 signal.

4 Cyclic voltammotry

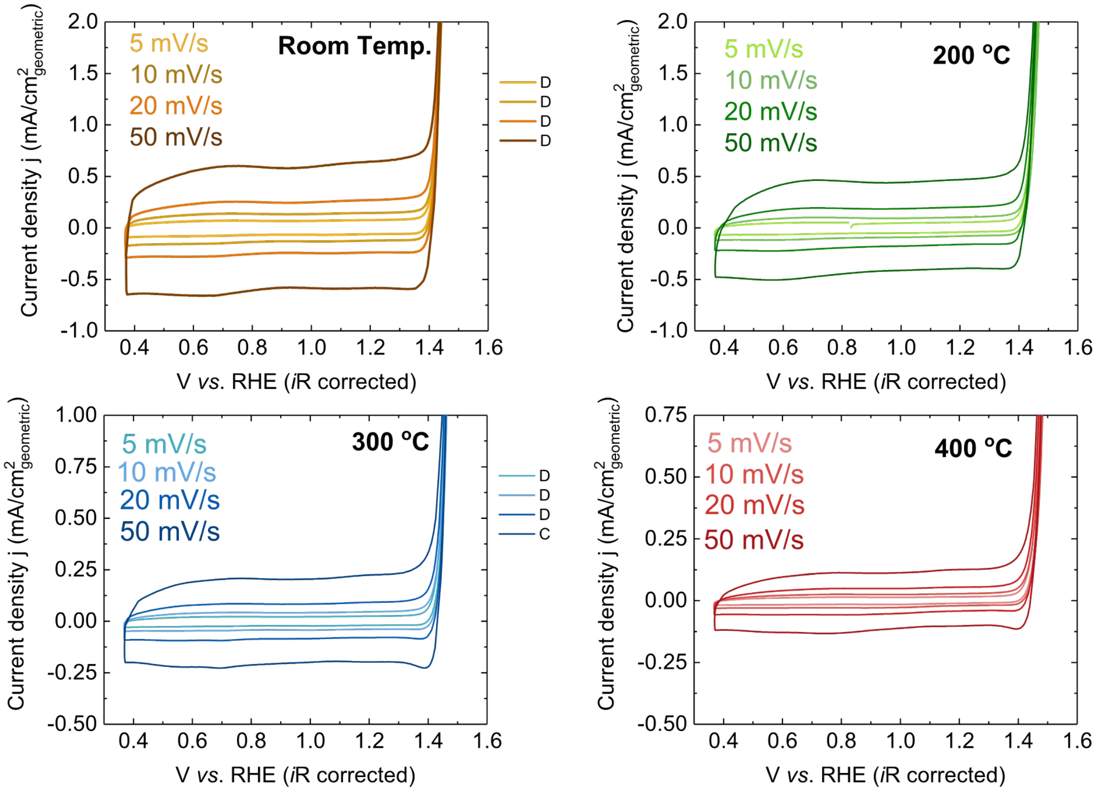

Figure 4: Cyclic voltammograms of RuOx sputtered films deposited at room temperature, 200°C, 300°C and 400°C. Measurements were made at scan rates of 5 mV/s, 10 mV/s, 20 mV/s and 50 mV/s in Ar-saturated 0.1 M HClO4 solution.

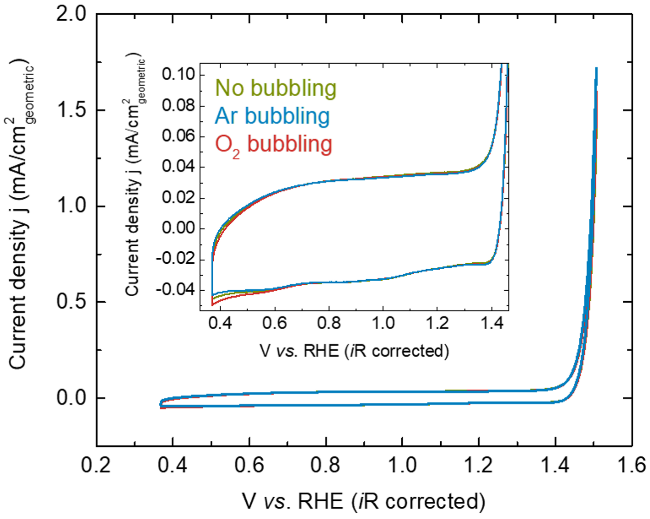

Figure 5: Cyclic voltammograms of RuOx sputtered films deposited at 400°C in electrolyte saturated with Ar (blue), O2 (red) and without any gas saturation (green). Measurements were made at scan rates of 10 mV/s in 0.1 M HClO4 solution.

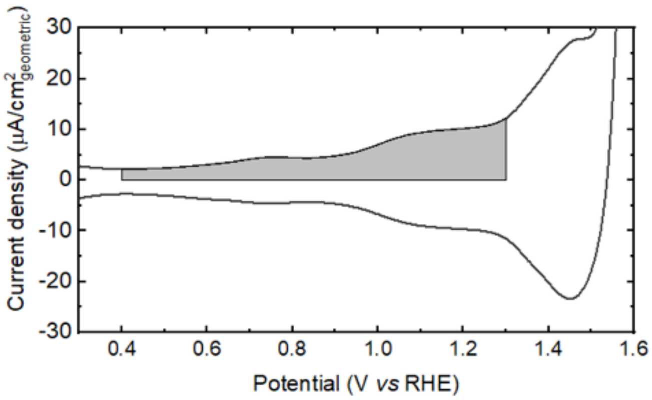

Figure 6: Cyclic voltammogram of a RuO2(110) single crystal at 50 mV/s in Ar-saturated electrolyte. The charge in to the shaded area corresponds to 105 µC/cm2 over 0.9 V, resulting in a capacitance of 115 µF/cm2.

5 Tafel regimes on sputter-deposited Ru films

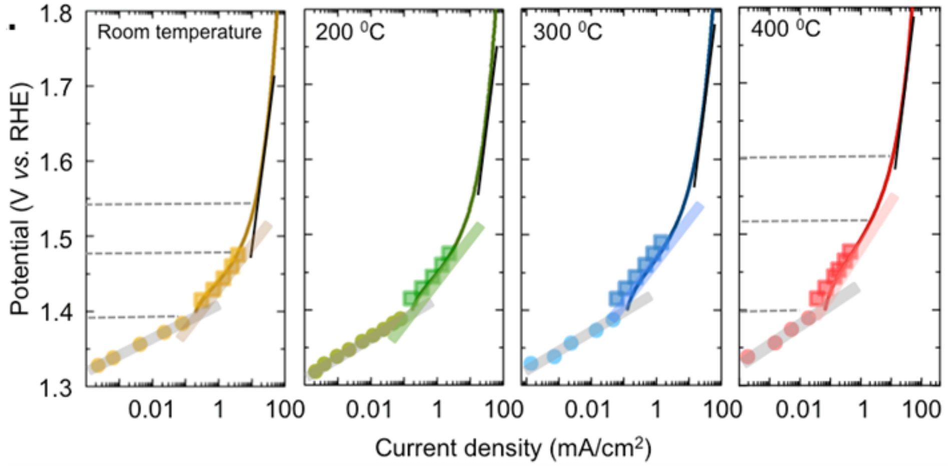

Figure 7: Tafel plot of RuOx surfaces prepared by sputtering at room temperature (in yellow), 200°C (in green), 300°C (in blue) and 400°C (in red). Two distinct changes in Tafel slope are observed. At 1.4 VRHE, the Tafel slope changes from 30 mV/decade to 60 mV/decade. The potential for the change in Tafel slope from 60 mV/decade is dependent on the sputtering temperature and increases from 1.47 VRHE on the room temperature sputtered film to 1.52 VRHE on the 400°C sputtered film. Filled circles represent the data obtained using mass spectrometry, filled squares represent the data obtained by potentiostatic measurements and the curve represents the capacitance-corrected cyclic voltammograms. Lines showing the three different Tafel slopes are drawn as a guide to the eye. Horizontal dotted lines are drawn to indicate the transition from one Tafel region to another.

6 Kinetic model

In this section of the SI, we outline the kinetic models used in main-text Figure 5b. First, below, we introduce the assumptions behind the most general form of the model's a rate equation, introduce all terms in the equation, and motivate the assumptions. Then we describe specific cases: (i) two sites with only one state each (Static coverage model, subsection 6.1), which cannot fit the our observations; and (ii) one site with multiple states (Potential-dependent coverage, subsection 6.2), which can. In subsection 6.4, we describe how the potential-dependent coverage enables an estimation of the energy of the surface states in the catalytic cycle, and describe our fitting procedure to do so.

If we can make the following assumptions about a multi-electron electrochemical reaction:

1. The back reaction is negligible
2. Mass transport is infinitely fast compared to the reaction kinetics
3. The coverage of different **sites** on the surface, defined as a unit of the surface that can catalyze the reaction by an unchanging mechanism, is constant, though the coverage of the **states** of each site may change with potential.
4. For each type of site, there is a single electron transfer step in the catalytic cycle, the **rate-determining step** (RDS), which is the bottleneck. All other steps are essentially at equilibrium.

Then the reaction rate as a function of potential can be described in general as[3]

$$j = \sum_{m}^{M} j^{\circ}_{m,\mathrm{RDS}}\, \theta_{m,\mathrm{RDS}}\, \exp\left(\frac{\alpha_{m,\mathrm{RDS}}\mathcal{F}(U - U^{\circ})}{RT}\right) , \tag{13}$$

where:

- j is the surface-normalized current density
- R is the gas constant
- T is the temperature (298.15 K)
- $\mathcal{F}$ is Faraday's constant
- U is the potential (E in the main text)
- U° is a reference potential (E° in the main text). We will use the standard equilibrium potential for the OER, $U^{\circ} = 1.23\,\mathrm{V_{RHE}}$.
- M is the number of different sites
- $\theta_{m,\mathrm{RDS}}$ is the coverage of the state of m from which the RDS takes place.
- $\alpha_{m,\mathrm{RDS}}$ is the **symmetry factor** of the RDS of site m. This is defined as the portion of $\mathcal{F}\mathrm{d}U$ by which the activation barrier for the step is lowered if the potential is increased by $\mathrm{d}U$.
- $j^{\circ}_{m,\mathrm{RDS}}$ is the **exchange current density** of the state of site m from which its rate determining step takes place. This defined as the surface-normalized current density at $U = U^{\circ}$ if the surface were fully covered by of the state of site m before its rate-determine step takes place, i.e $\theta_{m,\mathrm{RDS}} = 1$. This will in general be not only difficult to measure because it is a small current density, but impossible to measure directly because $\theta_{m,\mathrm{RDS}} < 1$ at $U = U^{\circ}$. It is thus in general a purely theoretical quantity.

Now, we motivate the validity of the assumptions above for our oxygen evolution experiments on RuO_2 at low overpotential.

1. The back reaction (ORR) is negligible, as the current density and O_2 production rate are identical in O_2-saturated electrolyte and He-saturated electrolyte (see Figures 2 and 5).

2. The mass transport processes in OER are protons away from the surface (4 per cycle), water to the surface (2 per cycle), and O_2 from the surface (1 per cycle). Of these, the transport of O_2 is by far the slowest, but because no difference in reaction rate was observed in O_2-saturated electrolyte (see again Figure 2), we conclude that mass transport does not influence the rate at the low current densities explored in this work.

3. The third assumption, that the abundance and mechanism of each type of site is unchanging, is a common assumption in microkinetic modeling[4, 5, 6, 7, 8, 9]. A motivation of this comes from the stability and repeatability of our activity measurements. The O_2 production rate at constant potential was constant over 15 hours, and the activity measured on the way up was identical to that measured on the way down during a potential staircase experiment (main-text Figure 2). We also argue under the static coverage model that a mechanism that changes with potential cannot by itself explain the data.

4. A single rate-determining step is likewise a common assumption in microkinetic modeling [4, 5, 6, 7, 8, 9], but worth discussing. The second part of this assumption, that the rate-determining step involves an electron transfer, is non-obvious, and is a topic of debate [10]. Both are discussed in more detail below. Briefly, the rate equation can still be approximated by Equation 13 if these assumptions do not hold: with multiple rate-determining step, the rate of each rate-determining step for a single site must be identical, and either could be used, with the caveat that $\theta_{m,\mathrm{RDS}}$ is not in equilibrium with all other θ's. If the RDS of a specific site is a purely chemical step, then $\alpha_{m,\mathrm{RDS}} = 0$ for that site, such that all of the potential dependence is in $\theta_{m,\mathrm{RDS}}$, but the form of Equation 13 still holds.

The goal of the model is to explain our data, wherein a range of RuOx catalysts show a common activity vs potential behavior, with a potential dependence that starts strong, at 25 mV/decade below 1.4 V vs RHE, and gradually weakens with increasing potential, to 120 mV/decade above 1.5 V vs RHE (main-text Figure 4, Figure S7).

6.1 Static coverage models

In a static coverage model, we assume that the rate-determining step takes place from a surface state which does not change in abundance as a function of potential. In other words the active states are static, and have constant coverage, i.e. $\theta_{m,\mathrm{RDS}} = \theta_m =$ constant.

One site:

If there is a constant coverage of the active surface species in its active state, then Equation 13 simplifies to:

$$j = j^\circ \exp\left(\frac{\alpha \mathcal{F}(U - U^\circ)}{RT}\right) \tag{14}$$

The potential dependence is most conveniently described after taking the logarithm, using base 10 by convention, of the current density normalized to the exchange current density:

$$\log\left(\frac{j}{j^\circ}\right) = \frac{\alpha \mathcal{F}(U - U^\circ)}{RT \ln(10)} \tag{15}$$

Note that the Nernst constant, $\mathcal{F}/(RT\ln(10))$, which is 60 mV at standard temperature, appears in the equation. The Tafel slope is defined as the reciprocal of the derivative of the base-10 logarithm with respect to potential, i.e. the potential change needed to achieve a factor-10 increase (a decade) in current density:

$$(\text{Tafel Slope})^{-1} = \frac{\partial \log(j/j^\circ)}{\partial U} = \frac{\alpha \mathcal{F}}{RT \ln(10)} \approx \frac{\alpha}{60\,[\text{mV}]} \tag{16}$$

The symmetry factor for simple electron transfer steps is typically 0.5[3], so, this results in a Tafel slope of 120 mV per decade, much higher than that observed. Symmetry factors are by their definition between 0 and 1, so the smallest Tafel slope possible with a single active state of constant coverage is 60 mV per decade. So even if the mechanism changes, a single-site static coverage model cannot explain a Tafel slope lower than 60 mV per decade.

Two sites:

We consider the activity originating from two sites, named such that site 1 has a larger coverage-weighted exchange current density than site 2, i.e.

$$j_1^\circ > j_2^\circ \tag{17}$$

The total current density is a superposition of the current density due to the two sites:

$$j = j_1 + j_2 = j_1^\circ \exp\left(\frac{\alpha_1 \mathcal{F}(U - U^\circ)}{RT}\right) + j_2^\circ \exp\left(\frac{\alpha_2 \mathcal{F}(U - U^\circ)}{RT}\right) \tag{18}$$

We now consider the three possible relationships between the symmetry factors below, i.e., $\alpha_1 = \alpha_2$, $\alpha_1 > \alpha_2$, and $\alpha_1 < \alpha_2$.

If the symmetry factors for the two current densities are equal, i.e. $\alpha_1 = \alpha_2$, then the terms can be grouped and it reduces to the form for a single site:

$$j = (j_1^\circ + j_2^\circ) \exp\left(\frac{\alpha \mathcal{F}(U - U^\circ)}{RT}\right) = j^\circ \exp\left(\frac{\alpha \mathcal{F}(U - U^\circ)}{RT}\right) \;, \;\; \text{if} \;\; \alpha_1 = \alpha_2 = \alpha \tag{19}$$

This would imply a constant Tafel slope and so cannot explain the data.

If the symmetry factors are not equal, then there is a potential at which the two sites in theory contribute equally to the overall current density, which we will call U_{cross}. By setting $j_1 = j_2$, we can solve for this potential:

$$U_{\text{cross}} = U^\circ + \frac{1}{\alpha_2 - \alpha_1} \ln\left(\frac{j_1^\circ}{j_2^\circ}\right) \tag{20}$$

If the site with the highest exchange current density also has the highest symmetry factor, i.e. $\alpha_1 > \alpha_2$, then $U_{\text{cross}} < U^\circ$ and the site with the higher exchange current density will dominate at all potentials higher than U°:

$$j \approx j_1^\circ \exp\left(\frac{\alpha_1 \mathcal{F}(U - U^\circ)}{RT}\right) \;\; \text{at} \;\; U > U^\circ \;, \;\; \alpha_1 > \alpha_2 \tag{21}$$

Finally, if one site has a higher exchange current density but a second site has a higher symmetry factor, then the dominant site switches at a potential U_{cross} which is greater than U°. Below this potential, the first site dominates:

$$\text{at} \quad U < U_{\text{cross}} \,, \quad \alpha_1 < \alpha_2 \tag{22}$$

$$j \approx j_1 = j_1^\circ \exp\left(\frac{\alpha_1 \mathcal{F}(U - U^\circ)}{RT}\right) \tag{23}$$

$$(\text{Tafel Slope})^{-1} = \frac{\partial \log(j/j^\circ)}{\partial U} = \frac{\alpha_1 \mathcal{F}}{RT \ln(10)} \tag{24}$$

Above U_{cross}, the second site dominates:

$$\text{at} \quad U > U_{\text{cross}} \,, \quad \alpha_1 < \alpha_2 \tag{25}$$

$$j \approx j_2 = j_2^\circ \exp\left(\frac{\alpha_2 \mathcal{F}(U - U^\circ)}{RT}\right) \tag{26}$$

$$(\text{Tafel Slope})^{-1} = \frac{\partial \log(j/j^\circ)}{\partial U} = \frac{\alpha_2 \mathcal{F}}{RT \ln(10)} \tag{27}$$

Since $\alpha_1 > \alpha_2$, the potential-dependence of $\log(j)$ *increases* as the potential increases past U_{cross}, i.e. the Tafel slope *decreases*. This is the opposite of what is observed in our experiments, where the lowest Tafel slope (strongest potential-dependence of $\log(j)$) is observed at low potentials and the Tafel slope increases at higher potentials.

This conclusion holds if there are more then two sites of static coverage on the surface, since any two of them can be labeled 1 and 2 and compared as above. Thus, we can rule out multiple sites of static coverage as an explanation of the changing Tafel slope we observe.

6.2 Potential-dependent coverage

Now, we consider the case where state of the active site, and thus the coverage of the active state, changes as a function of potential. We consider only one site for simplicity. Equation 13 reduces to:

$$j = j_{\text{RDS}}^\circ \theta_{\text{RDS}} \exp\left(\frac{\alpha_{\text{RDS}} \mathcal{F}(U - U^\circ)}{RT}\right) \tag{28}$$

Taking the logarithm, this becomes

$$\log\left(\frac{j}{j^\circ_{RDS}}\right) = \log(\theta_{\mathrm{RDS}}) + \frac{\alpha_{\mathrm{RDS}}\mathcal{F}(U-U^\circ)}{RT\ln(10)}\,, \tag{29}$$

and differentiating with respect to potential, the reciprocal Tafel slope is:

$$(\text{Tafel Slope})^{-1} = \frac{\partial \log\left(j/j^\circ_{\mathrm{RDS}}\right)}{\partial U} \tag{30}$$

$$= \frac{\partial \log(\theta_{\mathrm{RDS}})}{\partial U} + \frac{\alpha_{\mathrm{RDS}}\mathcal{F}}{RT\ln(10)} \tag{31}$$

$$= \frac{\partial \log(\theta_{\mathrm{RDS}})}{\partial U} + \frac{\alpha_{\mathrm{RDS}}}{60\,[\mathrm{mV}]} \tag{32}$$

The potential dependence now has two terms. One, $\alpha_{\mathrm{RDS}}/(60[\mathrm{mV}])$, is from the activation of the RDS, while the other, $\frac{\partial \log(\theta_{\mathrm{RDS}})}{\partial U}$, is due to the increasing coverage of the active state with potential.

Using a typical symmetry factor of 0.5 and the experimental Tafel slope, which at the lowest measured potentials is ≈ 24 mV/decade (rounding to the nearest divider of 120 mV/decade), we can solve for the potential dependence of the coverage of the active state:

$$\frac{\partial \log(\theta_{\mathrm{RDS}})}{\partial U} = (\text{Tafel Slope})^{-1} - \frac{\alpha_{\mathrm{RDS}}\mathcal{F}}{RT\ln(10)} \tag{33}$$

$$\approx \frac{1}{24\,[\mathrm{mV}]} - \frac{1}{120\,[\mathrm{mV}]} \tag{34}$$

$$= \frac{1}{30\,[\mathrm{mV}]} \tag{35}$$

In other words, at these low overpotentials, while it takes 120 mV to increase the rate of O_2 evolution normalized to the coverage of the active state by a factor of 10, it only takes 30 mV to increase the population of that state by a factor 10. Thus, according to this explanation, the coverage term is the most important for the overall potential dependence. We presented this analysis on a smaller data sets in refs [11, 9].

To elaborate on this explanation and determine if it can describe the *changing* Tafel slope in the experimental data, we develop an equation for θ_{RDS}. For this coverage to be changing, the site must be able to occupy one or more states. Specifically, we consider the state from which the rate-determining step takes place, or the **active state**, denoted $\mathrm{S}_{\mathrm{RDS}}$, a state less oxidized than this state by one electron, denoted $\mathrm{S}_{\mathrm{RDS-1}}$, and so on as $\mathrm{S}_{\mathrm{RDS}-n}$ up to $n = N$ electrons less oxidized than the rate-determining state (in practice we will use at most $N = 2$, or a total of three states). Each of the additional states can be oxidized to the active state according to the following reaction:

$$\mathrm{S}_{\mathrm{RDS}\text{-}n} \longrightarrow \mathrm{S}_{\mathrm{RDS}} + n\,(\mathrm{H}^+ + \mathrm{e}^-) \qquad \text{with} \qquad n \in [1, N] \tag{36}$$

Note that:

- $\mathrm{S}_{\mathrm{RDS}-n}$ with $n > 0$ can be but do not need to be part in the electrocatalytic cycle.
- While we have written Reaction 36 with an equal number of proton and electron transfers, pH-dependence studies would be needed to confirm this.

The equilibrium coverage of the states depends on their relative **standard free energies**. We define $\Delta^{\mathrm{RDS}}_{\mathrm{RDS}-n}G_0$ to be the standard free energy of the state n electrons reduced of the active state ($\mathrm{S}_{\mathrm{RDS}-n}$) relative to the active state ($\mathrm{S}_{\mathrm{RDS}}$). Here "standard", denoted by the subscript $_0$, means at idealized unity coverage, and should not be mixed up with the superscript $^\circ$, used to indicate the reference potential of $U^\circ = 1.23$ $\mathrm{V}_{\mathrm{RHE}}$. The standard free energy of a surface state relative to RDS can be expanded to make its potential dependence explicit:

$$\Delta^{\mathrm{RDS}}_{\mathrm{RDS}-n}G_0 = \Delta^{\mathrm{RDS}}_{\mathrm{RDS}-n}G^\circ_0 - n\mathcal{F}(U-U^\circ) \tag{37}$$

$$= \Delta_f G^\circ_0(\mathrm{S}_{\mathrm{RDS}}) - \Delta_f G^\circ_0(\mathrm{S}_{\mathrm{RDS}-n}) - n\mathcal{F}(U-U^\circ)\,, \tag{38}$$

where $\Delta_f G^\circ_0$ is the standard free energy of formation of the indicated state at idealized unity coverage at the reference potential. This is the quantity that can be calculated by density functional theory (DFT) given a guess at the structure of the surface state (on which which the present work does not speculate).

The standard free energy of S_{RDS-n} relative to S_{RDS} at the reference potential, $\Delta^{RDS}_{RDS-n}G^{\circ}_{0}$, is the quantity which will be used as a fitting parameter in the present work.

From the relative standard free energy, we can calculate an equilibrium constant K^{RDS}_{RDS-n}, which is the ratio of the coverage of S_{RDS-n} to the coverage of S_{RDS}. At the reference potential, this ratio is:

$$\left.\frac{\theta_{RDS-n}}{\theta_{RDS}}\right|_{U=U^{\circ}} = (K^{RDS}_{RDS-n})^{\circ} = \exp\left(-\frac{\Delta^{RDS}_{RDS-n}G^{\circ}_{0}}{RT}\right) \tag{39}$$

At other potentials, this coverage ratio is:

$$\frac{\theta_{RDS-n}}{\theta_{RDS}} = K^{RDS}_{RDS-n} = \exp\left(-\frac{\Delta^{RDS}_{RDS-n}G_{0}}{RT}\right) \tag{40}$$

$$= \exp\left(-\frac{\Delta^{RDS}_{RDS-n}G^{\circ}_{0} - n\mathcal{F}(U-U^{\circ})}{RT}\right) \tag{41}$$

$$= (K^{RDS}_{RDS-n})^{\circ} \exp\left(\frac{n\mathcal{F}(U-U^{\circ})}{RT}\right) \tag{42}$$

In the coming steps, it is more convenient to use its reciprocal,

$$\frac{\theta_{RDS}}{\theta_{RDS-n}} = K^{RDS-n}_{RDS} = \frac{1}{K^{RDS}_{RDS-n}} = (K^{RDS-n}_{RDS})^{\circ} \exp\left(-\frac{n\mathcal{F}(U-U^{\circ})}{RT}\right) . \tag{43}$$

Note that in the special case that $n = 0$, the state S_{RDS-n} is the same as S_{RDS} and this equilibrium constant K naturally becomes unity.

As such, assuming we have all states represented, we can compactly express the coverage of any state as:

$$\theta_{RDS-n} = \frac{K^{RDS-n}_{RDS}}{\sum_{i=0}^{N} K^{RDS-i}_{RDS}} = \frac{(K^{RDS-n}_{RDS})^{\circ} \exp\left(\frac{-n\mathcal{F}(U-U^{\circ})}{RT}\right)}{\sum_{i=0}^{N}(K^{RDS-i}_{RDS})^{\circ} \exp\left(\frac{-i\mathcal{F}(U-U^{\circ})}{RT}\right)} \tag{44}$$

The most interesting coverage for the OER is, of course, the coverage of the state from which the rate-determining step takes place. Its expanded expression is:

$$\theta_{RDS} = \frac{1}{\sum_{i=0}^{N}(K^{RDS-i}_{RDS})^{\circ} \exp\left(\frac{-i\mathcal{F}(U-U^{\circ})}{RT}\right)} \tag{45}$$

$$= \frac{1}{1 + (K^{RDS-1}_{RDS})^{\circ} \exp\left(\frac{-\mathcal{F}(U-U^{\circ})}{RT}\right) + (K^{RDS-2}_{RDS})^{\circ} \exp\left(\frac{-2\mathcal{F}(U-U^{\circ})}{RT}\right) + \ldots} \tag{46}$$

As such, we have all that is needed for modeling the OER kinetics. The fitting parameters are $\Delta^{RDS}_{RDS-n}G^{\circ}_{0}$ and j°_{RDS}. But how many values of n to include?

The analysis above of the lowest overpotentials can help. We determined that the potential dependence of the coverage of the active state should be a decade per 30 mV.

Differentiating Equation 46, let alone its logarithm, with respect to U would be difficult, so for now we make the simplification that one state, S_{RDS-n}, dominates the surface:

$$\theta_{RDS} \approx \frac{1}{(K^{RDS-n}_{RDS})^{\circ} \exp\left(\frac{-n\mathcal{F}(U-U^{\circ})}{RT}\right)} \qquad \text{if} \qquad \theta_{RDS-n} \approx 1 \tag{47}$$

This is much more wieldly. Taking the logarithm and differentiating by U, we get the potential dependence of the coverage:

$$\log(\theta_{RDS}) \approx \log\left((K^{RDS}_{RDS-n})^{\circ}\right) + \frac{n\mathcal{F}(U-U^{\circ})}{RT\ln(10)} \tag{48}$$

$$\frac{\partial \log(\theta_{RDS})}{\partial U} \approx \frac{n\mathcal{F}}{RT\ln(10)} \approx \frac{n}{60\,[\text{mV}]} \tag{49}$$

So the potential dependence of the active state is a decade for every 60 mV divided by the difference in oxidation between it and the state dominating the surface. Since we determined above that the potential dependence of the active state should be about a decade every 30 mV at the low overpotentials where it is steepest, $N = 2$ will suffice for fitting.

Inserting this into Equation 32 gives

$$(\text{Tafel Slope})^{-1} = \frac{(n + \alpha_{\text{RDS}})\mathcal{F}}{RT\ln(10)} \approx \frac{n + \alpha_{\text{RDS}}}{60\,[\text{mV}]}\,, \tag{50}$$

which motivates the definition of an effective symmetry factor α_{eff} (called $\alpha*$ in the main text), also called the **transfer coefficient**:

$$\alpha_{\text{eff}} = n + \alpha_{\text{RDS}}\,. \tag{51}$$

This is a specific case of a more general equation, presented elsewhere[12, 9] as

$$\alpha_{\text{eff}} = \frac{n_f}{\nu} + n_r\beta\,, \qquad \text{General coverage effect} \tag{52}$$

where n_f is the effective number of electron transfers to produce the active state; ν is the number of occurances of the RDS per catalytic cycle, here 1; n_r is the number of electrons transferred per RDS, here 1; and the symmetry factor of the RDS is called β.

Finally, we introduce $\langle n\rangle$, which is the effective average difference in oxidation between the active state and the surface:

$$\langle n\rangle = \sum_n n\,\theta_{\text{RDS}-n} = \frac{\sum_{n=0}^{N} n\,(K_{\text{RDS}}^{\text{RDS}-n})^\circ \exp\left(\frac{-n\mathcal{F}(U-U^\circ)}{RT}\right)}{\sum_{i=0}^{N}(K_{\text{RDS}}^{\text{RDS}-i})^\circ \exp\left(\frac{-i\mathcal{F}(U-U^\circ)}{RT}\right)} \tag{53}$$

This quantity, which can approximate n in Equation 50, decreases with increasing potential, so a potential-dependent coverage model of the active state is able to explain the decreasing potential dependence (increasing Tafel slope) observed.

6.3 Relative importance of coverage and driving force

In this subsection, we analyze the rate equation of the coverage dependent model derived above.

First, we expand Equation 28 by substituting Equation 46 for θ_{RDS} and, based on the observed Tafel slope, limiting the sum to three sites ($i \in [0, 1, 2]$), to get:

$$j = j_{\text{RDS}}^\circ \theta_{\text{RDS}} \exp\left(\frac{\alpha_{\text{RDS}}\mathcal{F}(U-U^\circ)}{RT}\right) \tag{54}$$

$$= j_{\text{RDS}}^\circ \frac{1}{1 + (K_{\text{RDS}}^{\text{RDS}-1})^\circ \exp\left(\frac{-\mathcal{F}(U-U^\circ)}{RT}\right) + (K_{\text{RDS}}^{\text{RDS}-2})^\circ \exp\left(\frac{-2\mathcal{F}(U-U^\circ)}{RT}\right)} \exp\left(\frac{\frac{1}{2}\mathcal{F}(U-U^\circ)}{RT}\right) \tag{55}$$

Then, we substitute Equation 39 for $(K_{\text{RDS}}^{\text{RDS}-i})^\circ$, regroup, and simplify the notation by introducing $G_i^\circ = \Delta_{\text{RDS}}^{\text{RDS}-i} G_0^\circ$, to get:

$$j = \frac{j_{\text{RDS}}^\circ \exp\left(\frac{\frac{1}{2}\mathcal{F}(U-U^\circ)}{RT}\right)}{1 + \exp\left(-\frac{G_1^\circ + \mathcal{F}(U-U^\circ)}{RT}\right) + \exp\left(-\frac{G_2^\circ + 2\mathcal{F}(U-U^\circ)}{RT}\right)} \tag{56}$$

This is the rate equation for an electrochemical reaction proceding from an active state in equilibrium with two more reduced states.

In the next section we use this rate equation to fit the experimental data. Here we comment on its characteristics.

At high overpotentials, the exponential terms in the bottom go quickly to zero and the potential dependence comes purely from the term in the top. This gives a Tafel slope of $2\frac{RT}{\mathcal{F}}\ln(10) = 120$ mV per decade. This is the contribution to the potential dependence of the driving force to the RDS, which is constant: **Driving the RDS or, equivalently, reducing its barrier, requires 120 meV improvement to increase activity by a factor of 10.**

The potential dependence of the denominator, on the other hand, is not constant. It changes directly with the coverage of the three states S_{RDS}, $S_{\mathrm{RDS}-1}$, and $S_{\mathrm{RDS}-2}$. At high overpotentials, as described in the last paragraph, the first term dominates and the denominator has no potential dependence. This corresponds to saturation of the surface sites with the active state, S_{RDS}. At low overpotential, the third term dominates, corresponding to coverage of the surface with $S_{\mathrm{RDS}-2}$. Under these conditions, the rate equation can be rewritten as

$$j = j^{\circ}_{\mathrm{RDS}} \exp\left(\frac{\frac{1}{2}\mathcal{F}(U-U^{\circ})}{RT}\right) \exp\left(+\frac{G^{\circ}_{2} + 2\mathcal{F}(U-U^{\circ})}{RT}\right), \qquad S_{\mathrm{RDS}-2}\ \text{dominant}. \tag{57}$$

The potential dependence of the second exponential factor is due to the effect of potential in bringing $S_{\mathrm{RDS}-2}$ up to S_{RDS}. Its contribution to the overall potential dependence corresponds to a Tafel slope of $\frac{1}{2}\frac{RT}{\mathcal{F}}\ln(10) = 30$ mV per decade. **Driving the population of the active state, or, equivalently, stabilizing it with respect to other surface states, only requires 30 meV improvement to increase activity by a factor of 10.**

This is an important point for electrocatalyst design.

An interesting related note is on the difference between potential-determining step and rate-determining step.

In the traditional metal-peroxide mechanistic model of oxygen evolution electrocatalysis, the scaling of the energies of the four surface states (*, *OH, *O, and *OOH) involved mean that in the best case two electron transfers or, more often, one electron transfer is much more uphill than the others, requiring higher potential to become downhill. A thermodynamic approach considers this last step to be come downhill the **potential determining step**.

On the other hand, a single rate-determing step means that when the free energies of the states of the electrocatalytic cycle are adjusted for the coverage of the intermediates, the entire excess energy input by the overpotential is released in a single step. This is how we sketched it in the inset to the lower panel of main-text Figure 5.

At the lowest potential that we were able to measure O_2 production, 1.29 $\mathrm{V_{RHE}}$, the excess free energy input is

$$\eta_{RDS}(1.29\,\mathrm{V_{RHE}}) = 4\mathrm{e}\cdot(1.29\,\mathrm{V} - 1.23\,\mathrm{V}) = 240\,\mathrm{meV} \tag{58}$$

per catalytic cycle. By the above reasoning, this entire excess free energy input goes to the RDS. Interestingly, this corresponds approximately to the expected limiting overpotential. But the coverage of the active state is minuscule. The reason much higher overpotential is required to get a substantial current density is that the vast majority of the extra driving force goes to populating the active state.

6.4 Fitting procedure for the coverage dependent model

As input to the fitting, we use experimental capacitance-normalized OER current (in amperes per farad, A/F) vs potential (in V vs RHE) for all sputter-deposited RuO_x (deposited at 25, 200, 300, and 400°C) and Ru foam samples. We use the O_2 signal measured by EC-MS below 1.45 V_{RHE} at all potentials where it was significantly different than the background signal. Higher potentials are excluded because of concerns about the potential distribution in the EC-MS cell at higher current densities. We use results from experiments in both $H_2{}^{18}O$ and $H_2{}^{16}O$ electrolytes. We add to this data set potential-hold activity measurements in the range 1.4 to 1.5 V vs RHE from RDE for RuO_x sputter-deposited at each temperature. All data points in this data set are weighted equally for the fit.

The experimental normalized current density are fit with the normalized current density calculated by Equation 28, the rate equation for one site under the assumptions at the start of this section. In its expanded form, it is:

$$j = \frac{j^{\circ}_{\mathrm{RDS}} \exp\left(\frac{\frac{1}{2}\mathcal{F}(U-U^{\circ})}{RT}\right)}{1 + \exp\left(-\frac{G^{\circ}_{1} + \mathcal{F}(U-U^{\circ})}{RT}\right) + \exp\left(-\frac{G^{\circ}_{2} + 2\mathcal{F}(U-U^{\circ})}{RT}\right)} \tag{59}$$

Three fitting parameters are used:

- j°_{RDS}, the hypothetical capacitance-normalized OER current at the reference potential ($U^{\circ} = 1.23\,[\mathrm{V_{RHE}}]$) given unit activity (full coverage) of the active state.
- $G^{\circ}_{1} = \Delta^{\mathrm{RDS}-1}_{\mathrm{RDS}} G^{\circ}_{0}$, the standard free energy difference at the reference potential ($U^{\circ} = 1.23\,[\mathrm{V_{RHE}}]$) from the active state to a state one electron less oxidized.
- $G^{\circ}_{2} = \Delta^{\mathrm{RDS}-2}_{\mathrm{RDS}} G^{\circ}_{0}$, the standard free energy difference at the reference potential ($U^{\circ} = 1.23\,[\mathrm{V_{RHE}}]$) from the active state to a state two electrons less oxidized.

Note that for this fitting we did not vary α_{RDS} but instead set it to 1/2.

In the last subsection, we motivated that additional sites more reduced than $\mathrm{S}_{\mathrm{RDS}-2}$ are not needed, based on the Tafel slope observed.

Fitting was done by square error minimization using the `minimize` function from the `optimize` module of the `scipy` python package. Since the behavior at small overpotentials is of as much interest as the behavior at high overpotentials, we minimize the square error $\log(j)$ instead of the square error on j. The function to minimize is:

$$\mathrm{Error}(j^{\circ}_{\mathrm{RDS}}, G^{\circ}_1, G^{\circ}_2) = \sum_i^I \left(\log(j_{i,\mathrm{exp.}}) - \log\left(j(U_i;\, j^{\circ}_{\mathrm{RDS}}, G^{\circ}_1, G^{\circ}_2)\right)\right)^2 \tag{60}$$

Where i indicates an experimental data point (U_i, $j_{i,\mathrm{exp.}}$).

The results of the optimization is:

$$j^{\circ}_{\mathrm{RDE}} = 9.4 \cdot 10^{-4}\,[\mathrm{A/F}] \tag{61}$$

$$G^{\circ}_1 = \Delta^{\mathrm{RDS}-1}_{\mathrm{RDS}} G^{\circ}_0 = -0.191\,[\mathrm{eV}] \cdot N_A = -18.4\,[\mathrm{kJ/mol}] \tag{62}$$

$$G^{\circ}_2 = \Delta^{\mathrm{RDS}-2}_{\mathrm{RDS}} G^{\circ}_0 = -0.325\,[\mathrm{eV}] \cdot N_A = -31.4\,[\mathrm{kJ/mol}] \tag{63}$$

where we have used Avogadro's number N_A to convert between kilojoules per mol of sites and electron volts per site (note that $\mathrm{e} \cdot N_A = \mathcal{F}$).

The root-mean-square error (in decades) is

$$\sqrt{\frac{\mathrm{Error}}{I}} = 0.35\,, \tag{64}$$

representing a factor of $10^{0.35} \approx 3$ spread in the combined set of activity measurements. While that may sound like a lot, note that at the lowest overpotentials, due to the very low Tafel slope this corresponds only to approximately ± 10 mV variation between samples.

With the fitting parameters solved, we can also describe the free energies of the states relative to the active state via Equation 37, the distribution of the states through Equation 44, and the effective number of electrons needed through Equation 53, all as a function of potential. These are included in the lower panels in main-text Figure 5.

6.5 Remarks on equilibrium

We now return to the concept of equilibrium, touched upon in Section 2 of this SI. There, we mentioned that the equilibrium potential depends on the O_2 concentration at the electrode surface according to the Nernst equation (eq. 1), and that the O_2 concentration at the electrode surface in EC-MS experiments is linearly related to the geometric O_2 production rate according to the mass transport of O_2 from the surface, equation 3. We can define the *Nernst overpotential* as the difference between this equilibrium potential and the applied (ohmic-drop-corrected) potential:

$$\eta_{\mathrm{Nernst}} = U - U_{\mathrm{eq}} = U - U^{\circ} - \frac{RT}{4\mathcal{F}} \ln(a^{\mathrm{O}_2})\,. \tag{65}$$

This is in contrast to the *nominal overpotential* referred to occasionally in the text, which ignores the Nernst shift and is simply the applied (ohmic-drop-corrected) potential in excess of $U^{\circ} = 1.23$ V vs RHE:

$$\eta_{\mathrm{nominal}} = U - U^{\circ} \tag{66}$$

In the EC-MS measurements, the only O_2 present is that generated by the electrode and removed through the chip, and the accessible potential range (avoiding bubbles) is that for which this is below saturation coverage, i.e. $a^{\mathrm{O}_2}_{\mathrm{ECMS}} < 1$. Thus, the Nernst overpotential is greater than the nominal overpotential. In the rotating disk electrode (RDE) chronoampermetric measurements included in the activity dataset, the electrolyte is saturated with O_2, so $a^{\mathrm{O}_2}_{\mathrm{RDE}} = 1$ and the Nernst overpotential is equal to the nominal overpotential.

Figure 8a-b show the activity results plotted vs overpotential. The faded symbols show the activity against nominal overpotential, and the solid symbols against Nernst overpotential. When, in Figure 8b, the results have been normalized to capacitance, they converge well against nominal overpotential, but not against Nernst overpotential. This result agrees with that in Figures 2 and 5, which show that the oxygen partial pressure does not affect activity. In other words, the OER in the systems studied here is *kinetically controlled* and not *equilibrium controlled.* For this reason, we refer prefer referring to the absolute potential on the RHE scale and not the overpotential. The question remains, ***why* doesn't the Nernst overpotential matter**? We'll now attempt to answer.

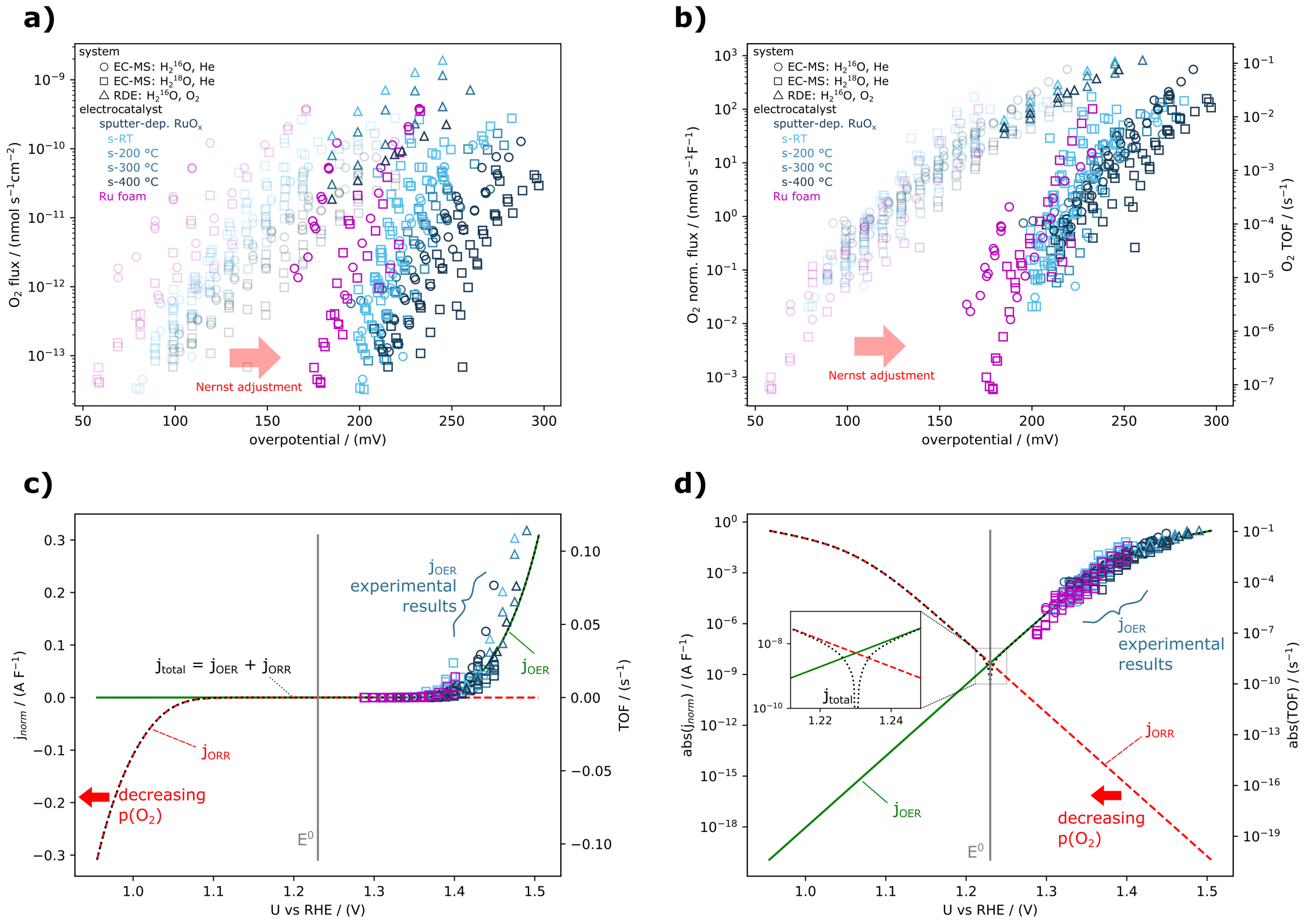

Figure 8: (a-b) Activity measurements plotted against nominal overpotential (faded symbols) and Nernst overpotential (solid symbols). The activity is taken to be O_2 production rate normalized to (a) geometric surface area or (b) capacitance. (c-d) Inclusion of the back reaction, oxygen reduction (ORR) in the model. (c) linear scale and (d) log scale. The modelled normalized OER current is green, the hypothetical normalized ORR current under 1 bar O2 in red dashed trace, and the hypothetical total current in black dotted trace. Note E is referred to as U in the SI text (please forgive us).

Equilibrium is defined as a set of conditions in which the forward and the backward reaction run at the same rate. For OER, the backwards reaction is the oxygen reduction reaction (ORR), $O_2 + 4\,(H^+ + e-) \longrightarrow 2\,H_2O$. The OER and ORR can be thought of as each having an independent current-vs-potential profile, and the equilibrium potential is where the currents associated with the two processes are of equal magnitude. Treating the forward and backward reactions as if they were elementary reactions (more on that below), the reason that the equilibrium potential shifts in a cathodic direction when p(O2) is decreased is a result of a change in ORR, not necessarily OER. This is illustrated in Figure 8c-d.

Here we assume an O_2-saturated electrolyte and assume that materials are equally good ORR catalysts as OER catalysts, i.e. we the magnitude of the ORR current at a given ORR overpotential is equal to the magnitude of the OER current at the same OER overpotential. Mathematically we assume that,

$$j_{\mathrm{ORR}}(U^\circ - \eta_{\mathrm{nominal}}) = -j_{\mathrm{OER}}(U^\circ + \eta_{\mathrm{nominal}})\,, \tag{67}$$

where η is the ORR overpotential and j_{OER} is assumed to be that predicted by the model, Equation 59, fit to the experimental data. Note that this is almost definitely a huge overestimate of the ORR current density, since in reality RuO_2 is a much worse ORR catalyst than OER catalyst. However, Equation 67 must hold at $U = 1.23\,, \mathrm{V_{RHE}}$, i.e. $\eta = 0$, because the second law of thermodynamics demands that there is no net activity at the equilibrium potential:

$$j_{\text{total}}(U^\circ) = j_{\text{ORR}}(U^\circ) + j_{\text{OER}}(U^\circ) = 0 \quad (68)$$

This assumption gives the symmetry around U° in Figure 8, but functions only as a conceptual help because j_{ORR} is only treated hypothetically. Nonetheless it makes clear that, in the potential range of our OER data, $U \geq 1.29$ V_{RHE}, the ORR current must be at least as small as the exchange current density, and thus several orders of magnitude smaller than the OER current density. This is in stark contrast to the case of hydrogen evolution on platinum, where there is a sizeable potential range over which both forward and backward reaction play a role. The inset of Figure 8d, hypothetical in our case, will look concretely familiar to an experimentalist who has worked with HER on Pt.

Because the ORR current is expected to be undetectably small at the potentials in which we are able to observe O2 generation (at 1.30 VRHE in O2-saturated electrolyte, we would predict the ORR current to be ten thousand times smaller than the OER current), the presence or absence of ORR, and thus the equilibrium potential, does not affect the measured OER activity. It also explains why the oxygen partial pressure does not effect the OER activity (Figures 2 and 5): Changing the O_2 partial pressure changes the ORR current, but this makes no difference to the total current where we can measure the OER rate.

The attentive SI reader might object at this point that we've just treated the OER as a single reaction, right after having broken it into steps - couldn't the O_2 partial pressure have an effect on the rate if it was involved in one of the elementary steps?

The answer to this must be yes - O_2 partial pressure would effect the rate if O_2 were involved in the equilibrium before the rate determining step. In other words, we sneakily assumed that Reaction 36 did not involve any O_2. The lack of dependence of rate on O_2 partial pressure would seem to confirm this. Like the lack of isotopic scrambling between dissolved O_2 and the oxygen in the water being oxidized (Section 2 of this SI), the observation is consistent with $^*OOH \longrightarrow O_2 + * + (H^+ + e^-)$ being the rate determining step.

Note that, unlike the O_2 partial pressure, the proton concentration *is* expected to influence the equilibrium of the steps up to the active state. Low-overpotential regime pH effects are therefore of high interest for future mechanistic investigations, but are out of the scope of the present work.

7 Database contents

This section shows figures of some of the datasets included in the database accompanying this article, available when this article is published at `https://github.com/ixdat/LowOverpotentialRegime`

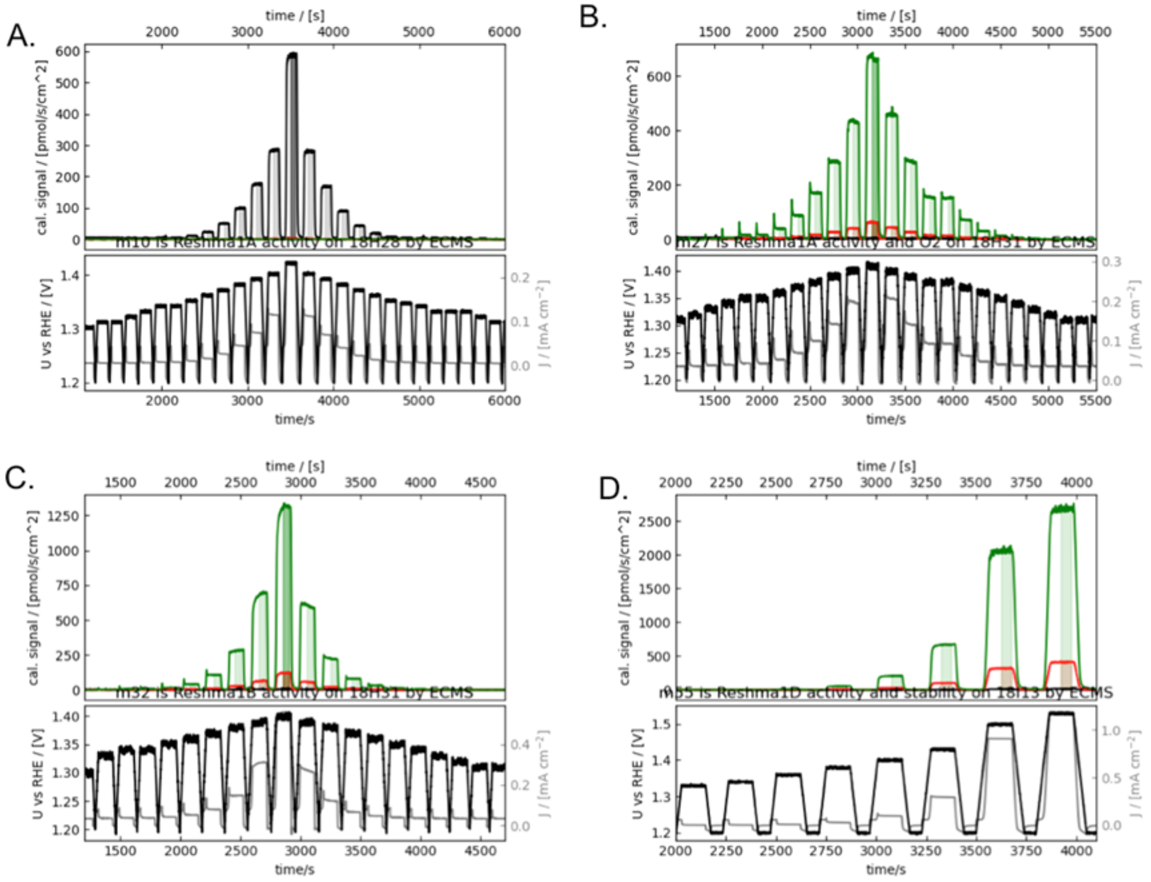

Figure 9: Four independent measurements for calibrated oxygen signals from OER on a RuOx film sputtered at room temperature for the three O2 isotopes in un-labeled electrolyte (m/z = 32 in grey, m/z = 34 in red and m/z = 36 in green). The lower half of each panel shows the potential applied in black and corresponding current measured in grey. Panel (A) shows data in unlabeled electrolyte, while Panel B-D show data in O-18 labeled 0.1 M HClO4.

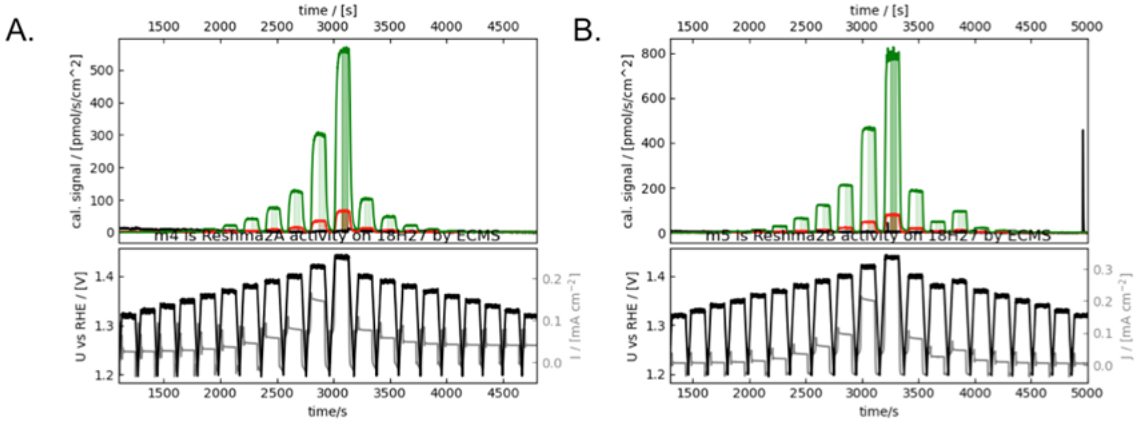

Figure 10: Four independent measurements for calibrated oxygen signals from OER on a RuOx film sputtered at 200°C for the three O2 isotopes in un-labeled electrolyte (m/z = 32 in grey, m/z = 34 in red and m/z = 36 in green). The lower half of each panel shows the potential applied in black and corresponding current measured in grey. Measurements were performed in O-18 labeled 0.1 M HClO4.

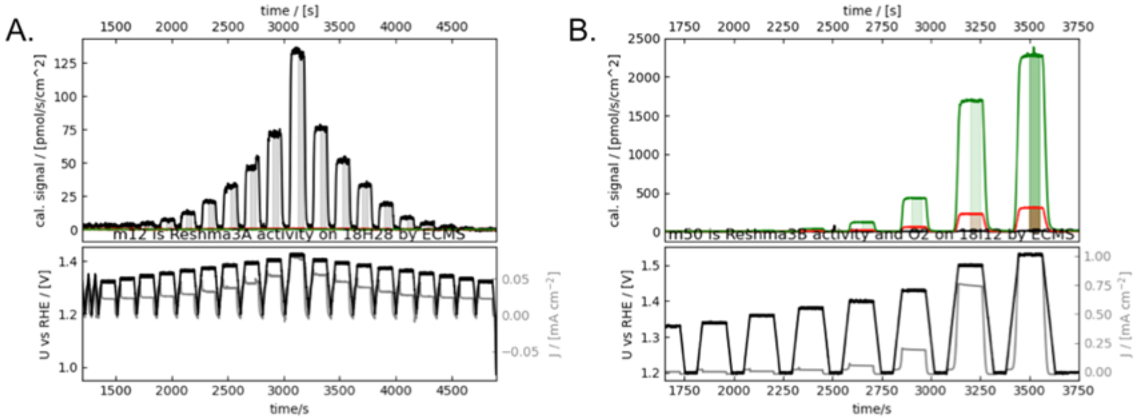

Figure 11: Four independent measurements for calibrated oxygen signals from OER on a RuOx film sputtered at 300oC for the three O2 isotopes in un-labeled electrolyte (m/z = 32 in grey, m/z = 34 in red and m/z = 36 in green). The lower half of each panel shows the potential applied in black and corresponding current measured in grey. Panel (A) shows data in unlabeled electrolyte, while Panel B show data in O-18 labeled 0.1 M HClO4.

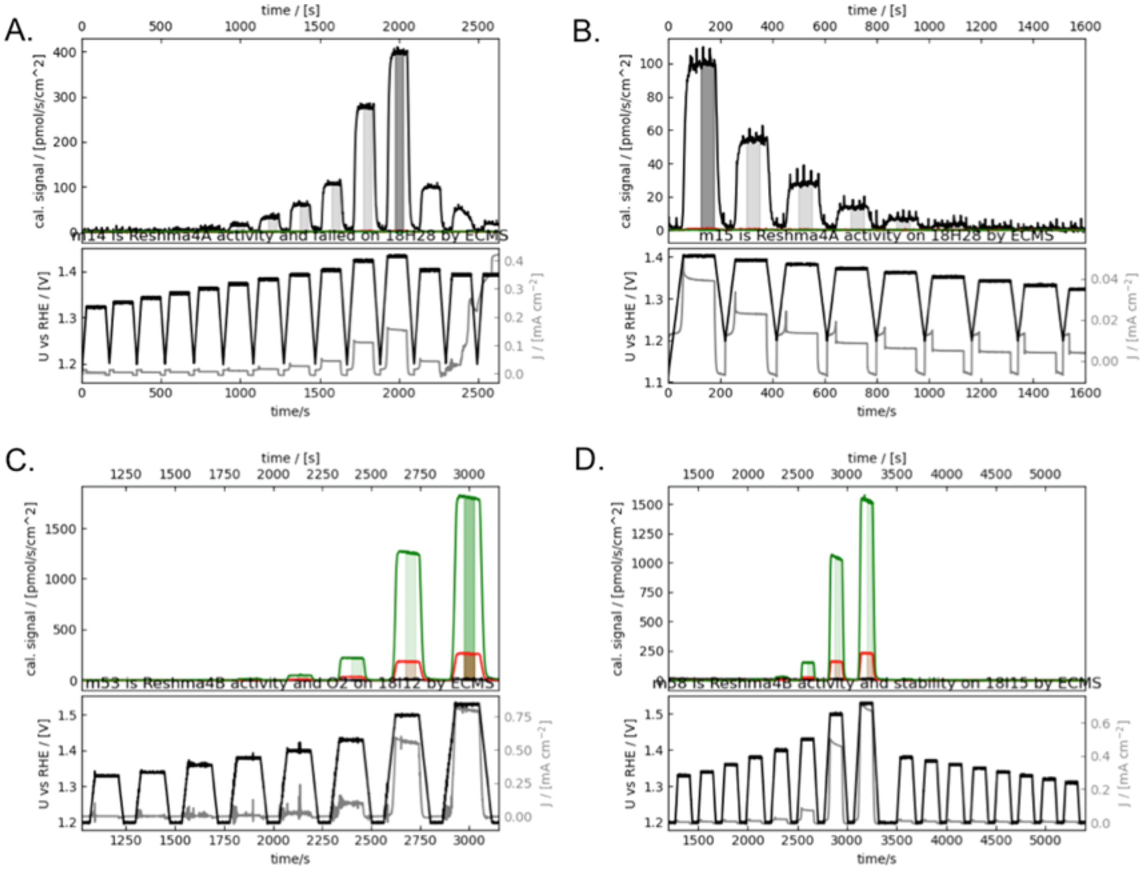

Figure 12: Four independent measurements for calibrated oxygen signals from OER on a RuOx film sputtered at 400oC for the three O2 isotopes in un-labeled electrolyte (m/z = 32 in grey, m/z = 34 in red and m/z = 36 in green). The lower half of each panel shows the potential applied in black and corresponding current measured in grey. Panel (A) and (B) show data in unlabeled electrolyte, while Panel (C) and (D) show data in O-18 labeled 0.1 M HClO4.

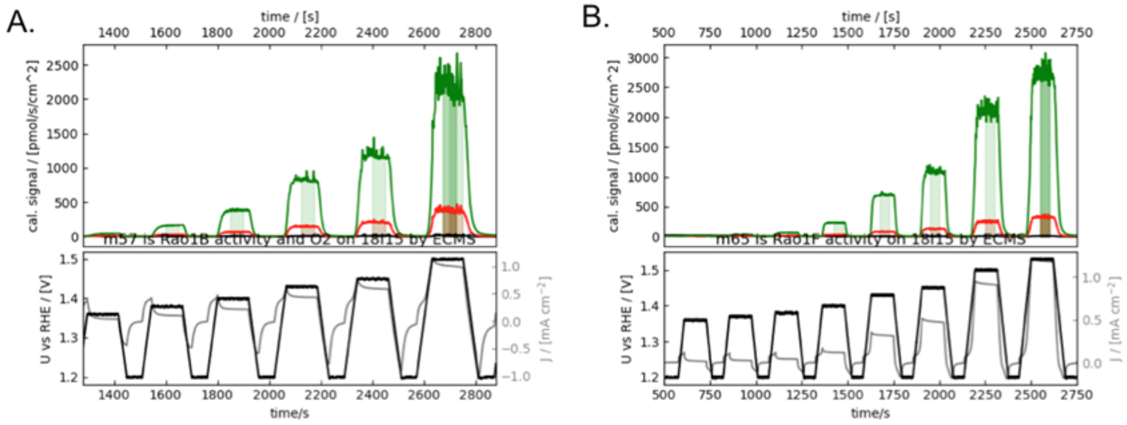

Figure 13: Two independent measurements for calibrated oxygen signals from OER on a RuOx nanoparticles for the three O2 isotopes in un-labeled electrolyte (m/z = 32 in grey, m/z = 34 in red and m/z = 36 in green). The lower half of each panel shows the potential applied in black and corresponding current measured in grey. Experiments were performed in in O-18 labeled 0.1 M HClO4.

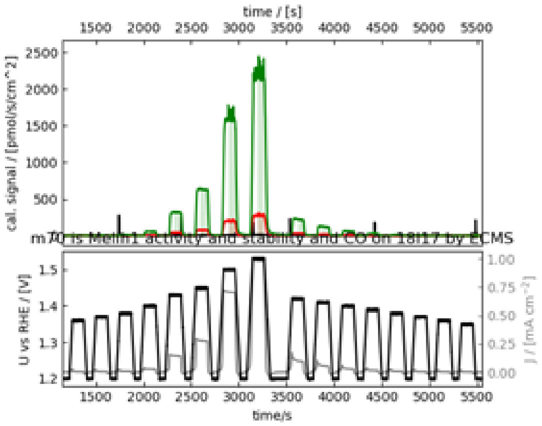

Figure 14: Measurement of calibrated oxygen signals from OER on a Ru film for the three O2 isotopes in un-labeled electrolyte (m/z = 32 in grey, m/z = 34 in red and m/z = 36 in green). The lower half of each panel shows the potential applied in black and corresponding current measured in grey. Experiments were performed in O-18 labeled 0.1 M HClO4.

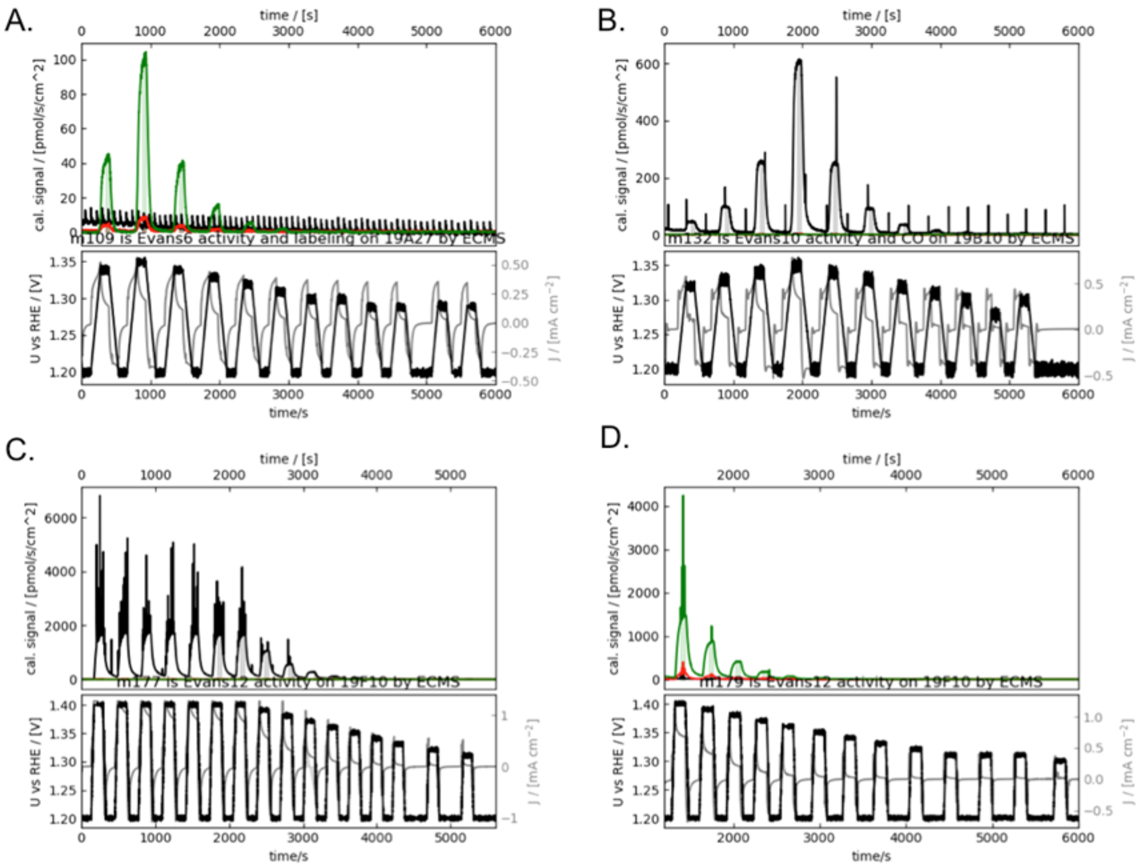

Figure 15: Four independent measurements for calibrated oxygen signals from OER on a RuOx foam for the three O2 isotopes in un-labeled electrolyte (m/z = 32 in grey, m/z = 34 in red and m/z = 36 in green). The lower half of each panel shows the potential applied in black and corresponding current measured in grey. Panel (A) and (D) show show data in O-18 labeled 0.1 M HClO4 while panel (B) and (C) show data in unlabeled electrolyte.

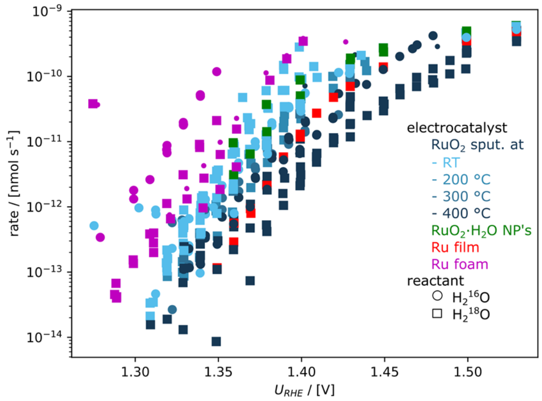

Figure 16: Experimentally measured oxygen flux for different RuOx sputtered films, hydrous oxide nanoparticles, Ru films and Ru foam.

References

[1] Daniel B. Trimarco, Soren B. Scott, Anil H. Thilsted, Jesper Y. Pan, Thomas Pedersen, Ole Hansen, Ib Chorkendorff, and Peter Vesborg. Enabling real-time detection of electrochemical desorption phenomena with sub-monolayer sensitivity. *Electrochimica Acta*, 268:520–530.

[2] Soren B. Scott, Jakob Kibsgaard, Peter C. K. Vesborg, and Ib Chorkendorff. Tracking oxygen atoms in electrochemical co oxidation – part i: Oxygen exchange via co2 hydration. *Electrochimica Acta*, 374, 2021.

[3] Allen J Bard and Larry R Faulkner. *Electrochemical methods: fundamentals and application.* Electrochemistry. I. Faulkner, Larry R. Wiley, New York, 2nd edition, 2001.

[4] J. O'M Bockris. Kinetics of activation controlled consecutive electrochemical reactions: Anodic evolution of oxygen. (24):817, 1956.

[5] Tatsuya Shinagawa, Angel T Garcia-Esparza, and Kazuhiro Takanabe. Insight on tafel slopes from a microkinetic analysis of aqueous electrocatalysis for energy conversion. *Scientific Reports*, 5:1–21, 2015.

[6] I. Chorkendorff and J. W. Niemantsverdriet. *Concepts of modern catalysis and kinetics.* Wiley-VCH, Weinheim, Germany, third, completely revised and enlarged edition. edition, 2017.

[7] Craig P. Plaisance, Simeon D. Beinlich, and Karsten Reuter. Kinetics-based computational catalyst design strategy for the oxygen evolution reaction on transition-metal oxide surfaces. *The Journal of Physical Chemistry C*, 123(13):8287–8303, 2018.

[8] J. Tyler Mefford, Zhenghang Zhao, Michal Bajdich, and William C. Chueh. Interpreting tafel behavior of consecutive electrochemical reactions through combined thermodynamic and steady state microkinetic approaches. *Energy and Environmental Science*, 13(2):622–634, 2020.

[9] Reshma R. Rao, Manuel J. Kolb, Livia Giordano, Anders Filsøe Pedersen, Yu Katayama, Jonathan Hwang, Apurva Mehta, Hoydoo You, Jaclyn R. Lunger, Hua Zhou, Niels Bendtsen Halck, Tejs Vegge, Ib Chorkendorff, Ifan E. L. Stephens, and Yang Shao-Horn. Operando identification of site-dependent water oxidation activity on ruthenium dioxide single-crystal surfaces. *Nature Catalysis*, 3(6):516–525, 2020.

[10] H. N. Nong, L. J. Falling, A. Bergmann, M. Klingenhof, H. P. Tran, C. Spori, R. Mom, J. Timoshenko, G. Zichittella, A. Knop-Gericke, S. Piccinin, J. Perez-Ramirez, B. R. Cuenya, R. Schlogl, P. Strasser, D. Teschner, and T. E. Jones. Key role of chemistry versus bias in electrocatalytic oxygen evolution. *Nature*, 587(7834):408–413, 2020.

[11] Soren B. Scott. *Isotope-Labeling Studies in Electrocatalysis for Renewable Energy Conversion and the Net CO2 Impact of this PhD Project.* Thesis, 2019.

[12] John O'M Bockris, Amulya K.N. Reddy, and Maria E. Gamboa-Aldeco. *Modern Electrochemistry 2A: Fundamentals of Electrodics.* Springer US, 2002.
